# Longitudinal Associations between Peer and Family Relationships, Emotional Symptoms, and Regional Brain Volume across Adolescence

**DOI:** 10.1007/s10964-023-01740-7

**Published:** 2023-02-20

**Authors:** Jessica Stepanous, Luke Munford, Pamela Qualter, Frauke Nees, Rebecca Elliott, IMAGEN Consortium, Tobias Banaschewski, Arun L. W. Bokde, Sylvane Desrivières, Herta Flor, Antoine Grigis, Hugh Garavan, Penny Gowland, Andreas Heinz, Rüdiger Brühl, Jean-Luc Martinot, Marie-Laure Paillère Martinot, Eric Artiges, Dimitri Papadopoulos Orfanos, Tomáš Paus, Luise Poustka, Sarah Hohmann, Sabina Millenet, Juliane H. Fröhner, Michael N. Smolka, Nilakshi Vaidya, Henrik Walter, Robert Whelan, Gunter Schumann

**Affiliations:** 1 Department of Psychology and Mental Health, University of Manchester, Manchester, Greater Manchester UK; 2 Division of Population Health, Health Services Research & Primary Care, University of Manchester, Manchester, Greater Manchester UK; 3 Manchester Institute of Education, University of Manchester, Manchester, Greater Manchester UK; 4 Department of Child and Adolescent Psychiatry and Psychotherapy, Central Institute of Mental Health, Medical Faculty Mannheim, Heidelberg University, Square J5, 68159 Mannheim, Germany; 5 Institute of Cognitive and Clinical Neuroscience, Central Institute of Mental Health, Medical Faculty Mannheim, Heidelberg University, Square J5, Mannheim, Germany; 6 Institute of Medical Psychology and Medical Sociology, University Medical Center Schleswig Holstein, Kiel University, Kiel, Germany; 7 Discipline of Psychiatry, School of Medicine and Trinity College Institute of Neuroscience, Trinity College Dublin, Dublin, Ireland; 8 Centre for Population Neuroscience and Precision Medicine (PONS), Institute of Psychiatry, Psychology & Neuroscience, SGDP Centre, King’s College, London, UK; 9 Department of Psychology, School of Social Sciences, University of Mannheim, 68131 Mannheim, Germany; 10 NeuroSpin, CEA, Université Paris-Saclay, F-91191 Gif-sur-Yvette, France; 11 Departments of Psychiatry and Psychology, University of Vermont, 05405 Burlington, VT USA; 12 Sir Peter Mansfield Imaging Centre School of Physics and Astronomy, University of Nottingham, University Park, Nottingham, UK; 13 Department of Psychiatry and Psychotherapy CCM, Charité – Universitätsmedizin Berlin, corporate member of Freie Universität Berlin, Humboldt-Universität zu Berlin, and Berlin Institute of Health, Berlin, Germany; 14 Physikalisch-Technische Bundesanstalt (PTB), Braunschweig and Berlin, Germany; 15 Institut National de la Santé et de la Recherche Médicale, INSERM U 1299 “Trajectoires développementales & psychiatrie”, University Paris-Saclay, CNRS; Ecole Normale Supérieure Paris-Saclay, Centre Borelli, Gif-sur-Yvette, France; 16 Ecole Normale Supérieure Paris Saclay, Centre Borelli, Gif-sur-Yvette, France; 17 Department of Child and Adolescent Psychiatry, Pitié-Salpêtrière Hospital, AP-HP. Sorbonne University, Paris, France; 18 Psychiatry Department, EPS Barthélémy Durand, Etampes, France; 19 Department of Psychiatry, Faculty of Medicine and Centre Hospitalier Universitaire Sainte-Justine, University of Montreal, Montreal, QC Canada; 20 Departments of Psychiatry and Psychology, University of Toronto, Toronto, ON Canada; 21 Department of Child and Adolescent Psychiatry and Psychotherapy, University Medical Centre Göttingen, von-Siebold-Str. 5, 37075 Göttingen, Germany; 22 Department of Psychiatry and Neuroimaging Center, Technische Universität Dresden, Dresden, Germany; 23 Centre for Population Neuroscience and Stratified Medicine (PONS), Department of Psychiatry and Neuroscience, Charité Universitätsmedizin Berlin, Berlin, Germany; 24 Centre for Population Neuroscience and Precision Medicine (PONS), Institute for Science and Technology of Brain-inspired Intelligence (ISTBI), Fudan University, Shanghai, China

**Keywords:** Latent change score model, Peer relationships, Family relationships, Adolescent mental health

## Abstract

The period of adolescence brings with it a dynamic interaction between social context and behaviour, structural brain development, and anxiety and depressive symptoms. The rate of volumetric change in the ventromedial prefrontal cortex (vmPFC) and amygdala have been implicated in socioemotional development in adolescence; typically, there is thinning of grey matter volume (GMV) in the vmPFC and growth in the amygdala during this time. The directionality of the associations between social, emotional, and neuroanatomical factors has yet to be untangled, such as the degree to which social variables impact regional brain development, and vice versa. To add, the differences between sexes are still up for debate. In this study, longitudinal associations between peer problems, family support, socioeconomic stress, emotional symptoms, amygdala volume, and vmPFC GMV were investigated for both sexes using latent change score models. Data from a multi-site European study at baseline (mean (SD) age = 14.40 (0.38) years; % female = 53.19) and follow-up 2 (mean (SD) age = 18.90 (0.69) years, % female = 53.19) were used. Results revealed that peer problems did not predict emotional symptoms, rather they changed together over time. For males only, there was positive correlated change between vmPFC GMV, peer problems and emotional symptoms, indicating that slower vmPFC GMV thinning was associated with poorer social and emotional functioning. Additionally, greater family support at age 14 years was associated with slower growth of amygdala volume between ages 14 and 19 years for males; previous research has related slower amygdala growth to resilience to mental health disorders. The findings have extended understanding of mutual social, emotional and brain development, and avenues to protect mental health.

## Introduction

Concerted changes in social and neuroanatomical factors in adolescence have implications for vulnerability and resilience to anxiety and depression. However, the directionality of associations between specific social, emotional, and neuroanatomical factors has yet to be untangled in adolescence, such as whether aspects of the peer and family environment are longitudinally associated with changes in emotional symptoms and brain structure. To add, differences between sexes in these associations have seldom been considered. Previous studies in the field are limited in (1) the failure to probe specific directional and associative relationships between variables over time in the same model, and (2) the lack of consideration of measurement error (Kline 2016; Könen & Karbach 2021). The current study addresses those problems by using latent change score modelling on a large adolescent dataset, to investigate the extent to which peer problems, family support, and socioeconomic stress predict emotional symptoms and the structural development of key brain regions of interest over time.

The social network widens from middle childhood (around 6 to 8 years old), with the focus shifting away from family relationships to peer relationships (Rueger et al. 2016). Peer relationships are particularly salient at this time, with peer problems and threats to peer group membership predictive of anxiety and depression symptoms (Parr et al. 2020; Rueger et al. 2016; Shin et al. 2016). At the same time, family support remains important during adolescence, and it is reported as the strongest predictor of mental health outcomes (Rothon et al. 2012; Rueger et al. 2008, 2016). Lack of parental warmth has been associated with greater psychological symptoms in adolescence, again underscoring the importance of positive parental behaviors in adolescent wellbeing (Muris et al. 2003). Overarching these interpersonal relationships is family socioeconomic status (SES; Bai et al. 2021), where low SES has been associated with lack of family support (Devenish et al. 2017). The family stress model explains these associations in terms of increased stress, resulting in lower parental support and reduced involvement with the child, with subsequent increases in child emotional problems (Conger & Conger, 2002; Conger & Donnellan, 2007). One remaining question is whether stress from SES affects the child’s relationships outside of the family, for which there is some evidence (Devenish et al., 2017). In one study, the child’s perceived stress found to mediate the relationship between SES and peer relationships, which again suggests that increased stress extends to other adolescent relationships (Bai et al., 2021). Thus, consideration of socioeconomic stress, family support and peer relationships together are needed to determine how these aspects of the social environment affect each other, and to establish which factors are the strongest predictors of emotional problems in adolescence.

Together with social factors, the brain undergoes rapid development in adolescence. The dual-systems model of brain development states that affective subcortical regions mature earlier than higher-level frontal regions in adolescence, which has implications for control over socioemotional processes and vulnerability to mental health problems (Blakemore 2008; Nelson et al., 2016). Subcortical regions such as the amygdala increase in volume from late childhood to late adolescence (age 16 years) before stabilising in the early 20s (Mills et al., 2014; Wierenga et al., 2014). On the other hand, the prefrontal cortex (PFC) decreases in grey matter volume (GMV) from early adolescence into the early 20s (Mills et al., 2014). This reduction in GMV is attributed to synaptic pruning to increase neural efficiency and refine cognitive control functions (Blakemore, 2008). This has been demonstrated in a specific region of the PFC known as the ventromedial prefrontal cortex (vmPFC), which has previously been defined as the anterior PFC, including the medial and orbital frontal cortex (mOFC; Hiser & Koenigs, 2018). Accelerated thinning in the vmPFC has been associated with fewer anxiety and depression symptoms in adolescence (Ducharme et al., 2014). In contrast, slower growth of the left amygdala was associated with resilience to psychopathology between early and mid-adolescence (Whittle et al., 2013). When investigated together, maturational coupling of less growth in amygdala volume and greater thinning in the anterior PFC (including vmPFC) was associated with fewer depressive symptoms across adolescence (Vijayakumar et al., 2017). This shows that the pattern of development within these regions is predictive of mental health problems, and adolescence could reflect a sensitive period that lays the foundation for a person’s social and emotional trajectory throughout their life course (Lamblin et al., 2017).

The connection between brain structure and socioemotional experiences is not deterministic; social experiences also shape the structure of the brain. Social network size has been positively associated with GMV in regions involved in emotional and social processing, including the amygdala and vmPFC/mOFC (Noonan et al., 2018). Social experiences have also been found to predict the developmental trajectory of socioemotional brain regions in adolescence. Increased adolescent social stress was associated with smaller decreases in GMV in prefrontal regions including the vmPFC/mOFC (Tyborowska et al., 2018). To add, positive parenting—defined as happy, validating and affectionate behavior during a family interaction assessment—has been associated with attenuated growth of the amygdala for boys and accelerated thinning of the mOFC for both sexes (Whittle et al., 2014). Left mOFC GMV has also been negatively associated with peer problems for both sexes (Kelly et al., 2015), although this study was cross-sectional and focused on childhood maltreatment. Altogether, it is unclear the degree to which socioeconomic stress, family support, and peer problems predict the structural development of the amygdala and vmPFC/OFC when considered together, and whether there are specific effects due to sex.

As alluded to in the previous point, sex is another factor that influences both neural development and social experiences. In terms of structural brain development, there is evidence that female brains mature faster than males’, with GMV peaking earlier and increasing more rapidly in females compared to males in regions including the amygdala (Goddings et al., 2014). This may explain neuroimaging findings mentioned previously, such as the relationship between positive parenting and slower growth of the amygdala for males in early adolescence (Whittle et al., 2014). Further, there are differences in social experiences between sex, with males more likely to interact with peers in larger groups (Rose & Rudolph, 2006), experience a range of peer victimization (Wang et al., 2010), and have less friend support compared to females (Rueger et al., 2008). These differences in social exposure/intimacy between the sexes could present unique opportunities where social experiences affect sensitive periods of brain development.

Additional factors other than peer and family relationships have been associated with both internalizing symptoms and structural brain development. Early stressful life events have been associated with changes in brain volume, emotional symptoms, and social functioning (Gorka et al., 2014; Hanson et al., 2010). To add, pubertal status varies between person in adolescence, and has been implicated in brain development (Giedd et al. 2006) and symptoms of anxiety and depression (Huerta and Brizuela-Gamiño, 2002). Whole brain volume has also been shown to differ between sexes (Kaczkurkin et al., 2019), and thus must be included to compare sex differences in regional brain volume. Psychiatric diagnosis also affects social and emotional functioning directly and through stigma (Kaushik et al., 2016), and recurrent emotional problems has been associated with regional changes in GMV, including the amygdala and frontal lobe (Bora et al., 2012). To add, the effects of location and recruitment center must be considered in multi-center studies, particularly due to potential variability between MRI scanners (Schumann et al., 2010). Thus, these variables these must be included in a model to account for confounding effects when assessing the link between social, emotional and neuroanatomical factors.

## Current study

Questions remain regarding the links between different aspects of the social environment—peer problems, family support, and socioeconomic stress—and emotional symptoms, whether these social factors together predict amygdala and vmPFC structural development, whether both amygdala and vmPFC development is associated with emotional symptoms, and whether these associations differ between sexes. These ideas have been explored in separate studies, but a model that considers these aspects together is lacking. The current study filled that gap using a multi-center European dataset—IMAGEN—and by applying latent change score modelling to test the direction of relationships of interest whilst accounting for measurement error. The hypotheses were based on research outlined previously; the peer and family environment predicted emotional symptoms and structural developmental trajectory of the amygdala and vmPFC, i.e. negative social experiences predicted greater increase of the amygdala and smaller decrease of the vmPFC over time. Greater peer problems at age 14 years will predict a larger increase in emotional symptoms between age 14 and 19 years for both sexes (Hypothesis 1). Greater peer problems at age 14 years will predict a larger increase in amygdala volume and smaller decrease in vmPFC GMV between age 14 and 19 years for both sexes (Hypothesis 2). Larger increases in amygdala volume and a smaller decrease in vmPFC GMV will mediate the relationship between higher peer problems and a larger increase in emotional symptoms between age 14 and 19 years for both sexes (Hypothesis 3). Higher family support at age 14 years will predict a decrease in peer problems and emotional symptoms between age 14 and 19 years for both sexes. Higher family support will also predict a smaller increase in amygdala volume for males only and a larger decrease in vmPFC GMV for females only, due to differences in normative structural brain development between sexes (Hypothesis 4). Higher family socioeconomic stress at age 14 years will predict an increase in peer problems and emotional symptoms between age 14 and 19 years for both sexes. Higher family socioeconomic stress will also predict a larger increase in amygdala volume for males and a smaller decrease in vmPFC GMV for females, due to differences in normative structural brain development between sexes (Hypothesis 5).

## Methods

### Participants

Data from participants in the IMAGEN project were used (https://imagen-project.org/). IMAGEN is a European multicenter study that contains biological, psychological, and environmental variables to assess development and behaviour in adolescence (Schumann et al., 2010). Participants were recruited from a diverse range of high schools across eight European sites (Dresden, Berlin, Mannheim, and Hamburg in Germany; London and Nottingham in the U.K.; Dublin in Ireland; and Paris in France). IMAGEN recruitment focused on diversity in socioeconomic status, academic achievement and behavioral/emotional functioning, and recruited people of European descent for homogeneity in the genetic analyses (Schumann et al., 2010). Local ethics research committees approved the study at each site and procedures were in accordance with the 1964 Declaration of Helsinki and its later amendments or comparable ethical standards. Written informed consent was obtained from all legal guardians.

The current analysis used data from participants who attended both the baseline (age 14 years; 2010) and follow-up 2 (age 19 years; 2015) assessments. Data from follow-up 1 (age 16 years; 2012) was not used in the current analysis as several measures were not available due to a lack of neuroimaging assessment. Of 2315 participants who had data from any variable of interest, 957 participants were included in the final sample. The reasons for removal of data were as follows: data quality issues identified by IMAGEN (*n* = 13, 0.6%), significant Mahalanobi’s distance outliers in the neuroimaging variables to account for scanning errors (*n* = 32, 1.4%), and complete data not available in variables of interest (*n* = 1313, 56.72%). The most common missing data patterns included missing all variables of interest at age 19 years (*n* = 532, 22.98%), missing Life Events Questionnaire data between 14 and 19 years (*n* = 173, 7.47%), all data missing except for ID, sex, and recruitment center (*n* = 66, 2.85%), and missing Childhood Trauma Questionnaire data at age 19 years (*n* = 58, 2.51%). Differences between the sample where data quality issues were removed (*n* = 2302) and the complete-case sample (*n* = 957) are described in Online Resource 1.

### Measures

Data at age 14 and 19 years were available for peer problems, emotional symptoms, and regional brain volumes. Only data at age 14 years were available for family support and family socioeconomic stress.

Items from the peer problems and emotional symptoms scales were used to create latent variables. For regional brain volume, left and right volumes were used to create latent variables (left and right amygdala volume, and left and right vmPFC GMV).

#### Peer problems

Peer problems were measured using the peer relationship problems section of the child-reported Strengths and Difficulties questionnaire (SDQ; Goodman, 1997). Participants responded to items such as being alone, being liked by peers, and being bullied over the last six months using a three-point Likert scale. There were slight differences in the wording of the questions between the versions used for age 14 and age 19 years (see Online Resource 2). The internal consistency of the peer problems scale has yielded Cronbach’s alpha values ranging from 0.15 (Essau et al., 2012) to 0.64 (Van Roy et al., 2008). To further assess scale reliability, coefficient omega values were calculated, which is suggested to be more robust method when using latent variables with ordinal indicators (Flora, 2020). Coefficient omega values were as follows: age 14 years male = 0.575, females = 0.525; age 19 years males = 0.444, females = 0.412, which shows that 41–58% of total score variance was due to the latent variable.

#### Emotional symptoms

Emotional symptoms were measured using the emotional symptoms section of the child-reported SDQ (Goodman, 1997). Participants noted the degree to which they had experienced various emotional symptoms such as somatic pains, worrying, and unhappiness in the last six months using a three-point Likert scale. There were slight differences in the wording of one question between the versions used for age 14 and age 19 years (see Online Resource 2). The internal consistency of the emotional symptoms subscale has previously ranged between acceptable and good values Cronbach’s alpha ranging from 0.61 (Van Roy et al., 2008) to 0.78 (Yao et al., 2009). Coefficient omega values were as follows: age 14 years male = 0.614, females = 0.588; age 19 years males = 0.730, females = 0.717, which shows that 59–73% of total score variance was attributed to the latent variable.

#### Family support

Family support was measured using the total score of the affirmation section of the parent-reported Family Life Questionnaire (FLQ; Last et al., 2012). Parents answered on a four-point Likert scale the degree to which their child gets love and affection, gets help and support when stressed, is praised and rewarded, and is liked and respected. These were then summed to produce a total score. A total score was used instead of a latent variable to reduce model complexity and increase rates of convergence. The FLQ affirmation scale has demonstrated satisfactory internal reliability (Cronbach’s alpha = 0.64–0.70; Last et al., 2012).

#### Family socioeconomic stress

Socioeconomic stress was measured by the total score of parent-reported socioeconomic/housing section of the Family Stresses Scale from the parent-reported Development and Well-Being Assessment (DAWBA; Goodman et al., 2000). Parents stated the degree to which unemployment, financial difficulties, home inadequacy, and neighbor problems made family life stressful, using a three-point Likert scale. As with the family support measure, a total score was produced by summing scores together to improve model convergence. Previous research has reported the Family Stresses Scale to have acceptable internal consistency (Cronbach’s alpha = 0.69; Lium, 2017).

#### Regional brain volume

Amygdala volume and vmPFC GMV were regions of interest in the present study due to their structural and functional significance in emotion and social relationships in previous studies (Hiser & Koenigs, 2018; Kim et al., 2011). T1-weighted images were processed by IMAGEN using FreeSurfer 5.3.0, to automatically parcellate the brain (Schumann et al. 2010). Amygdala volume comprised left and right amygdala volume from the Aseg atlas (Fischl et al., 2002). The vmPFC was defined as the combination of left and right medial orbitofrontal cortex GMV using the Desikan-Killiany atlas (Desikan et al., 2006), in line with previous studies (e.g. Powers et al., 2017). Inspection of the raw data showed significant negative skew, particularly in the vmPFC areas, which is prone to signal dropout (Juchem et al., 2010). Therefore, to account for potential errors in the neuroimaging data, multivariate outliers were identified and removed using Mahalanobi’s Distance (*n* = 32), as previously outlined. In the statistical models, amygdala volume and vmPFC GMV values were scaled (values divided by 1000), so that the values were closer in magnitude to other variables to allow for model convergence.

#### Covariates

Covariates in the models at age 14 years included recruitment center, psychiatric diagnosis, mean Pubertal Development Scale (PDS) score, total negative life events before age 14 years, and whole brain volume (WBV). Also included were total negative life events between age 14 and 19 years, and childhood trauma measured at age 19 years.

##### Recruitment center

Recruitment center was added as a dummy-coded covariate (reference category = Berlin).

##### Psychiatric diagnosis

Psychiatric diagnosis was a dummy-coded binary variable (reference category = no) determined from any DSM-IV or ICD-10 diagnosis from the DAWBA clinical rater. The clinical rating system has shown satisfactory inter-rater reliability (kappas ~0.70; Goodman et al. 1996).

##### Pubertal development

The PDS is a self-report measure of physical changes as a result of puberty, such as changes in height, body hair and skin, as well as male/female specific items (Carskadon & Acebo, 1993). Cronbach’s alpha for PDS items has previously ranged between 0.67 to 0.70 (Carskadon & Acebo, 1993).

##### Whole brain volume

WBV was measured by the BrainSegVolNotVent variable in Freesurfer. As with the amygdala volume and vmPFC GMV, WBV was scaled (values divided by 1000000) before entering it into the statistical models, to ensure variance was similar between variables.

##### Negative life events

Total negative life events were measured using the Life Events Questionnaire (Newcomb et al., 1981). Participants reported whether they had experienced 39 life events from seven scales, the age it happened, and the perceived valence of the event. Only events reported as negative were included and summed. A total score for negative life events prior to age 14 years was calculated using data from baseline (age 14 years); another total score for between ages 14 and 19 years was calculated using data from follow-up 1 (age 16 years) and follow-up 2 (age 19 years). Cronbach’s alpha for the LEQ scales has previously ranged between 0.36 and 0.58 (Newcomb et al., 1981).

##### Childhood trauma

Childhood trauma was measured using the Childhood Trauma Questionnaire (CTQ; Bernstein et al., 1994). This includes items related to experiences of physical and emotional abuse, emotional neglect, sexual abuse and physical neglect using a 5-point Likert scale. The current study used the total CTQ score obtained at follow-up 2 (age 19 years) as an index of abuse and neglect in childhood. Cronbach’s alpha ranged from 0.79 to 0.94, indicating high internal consistency (Bernstein et al.,^1994^).

### Statistical Approach

Analyses were conducted in R version 4.1.1 (R Core Team, 2012) using the lavaan package (Rosseel, 2012). Weighted least squares mean- and variance-adjusted (WLSMV) estimation was used for all analyses due to the presence of ordinal and categorical data (Brown, 2015). The standardized parameter estimates were reported using “std.all” in lavaan, which standardizes both the latent and observed variables (Rosseel 2022).

#### Measurement Invariance

Measurement invariance tests were conducted to assess whether the same constructs were measured between sex and over time. Separate confirmatory factor analysis models were used to test invariance of the peer problems and emotional symptoms latent variables across two time points (age 14 and 19 years) and between sex (male and female).

First, the configural model specified the latent variables at age 14 and age 19 years, and freely estimated the item loadings, thresholds, and residual covariance. The latent and item variables’ means/intercepts were fixed to 0 and variance fixed to 1 for model identification (Wu & Estabrook, 2016). The following constraints were then tested in sequential models: sex and time equivalence of factor loadings, item intercepts, and residual variances (Wu & Estabrook, 2016).

If there were no significant changes in fit when applying successive constraints, full invariance was achieved. If there were significant changes in fit, partial invariance was tested by investigating the modification indices to determine which parameter to free, if it was theoretically justified. The adjusted model was than compared to the previous best-fitting model, and parameters were sequentially freed until good model fit was achieved. For comparison of factor means to be valid, equivalence of thresholds, loadings and intercepts must be established at a minimum.

#### Latent change score models

Latent change score models (LCSM; McArdle & Hamagami, 2001) were used to test hypotheses about individual change and the interplay between peer problems, emotional symptoms, amygdala volume and vmPFC GMV between age 14 and 19 years. The same approach was used to investigate how cross-sectional family support and family socioeconomic stress at age 14 years were related to longitudinal change in the above variables.

LCSMs are a class of structural equation models that specify change as a latent variable, and thus account for measurement error in the observed difference between time points (Kievit et al., 2018). This approach brings with it the benefit of access to both group- and individual-level change parameters: group-level average change over time, individual variability in change, and the degree to which baseline values are related to the rate of change, all whilst factoring in baseline covariance (Kievit et al., 2018). LCSMs also allow for testing the proposed direction of relationships between variables over time. For example, one can test whether baseline peer problems affect change in emotional symptoms, whether baseline emotional symptoms affects change in peer problems, or whether there is correlated change between variables over time (Kievit et al., 2018). Furthermore, sex differences can be tested by fixing parameters to equality by sex and assessing changes in comparative model fit; significant changes in model fit provide evidence for sex differences.

First, univariate LCSMs were constructed to quantify within-variable change for peer problems, emotional symptoms, amygdala volume and vmPFC GMV. The feedback parameter was specified as covariance rather than a regression parameter to interpret raw change scores. Guided by the measurement invariance results, invariant parameters were constrained to equality within the model, whilst non-invariant items were freed.

To estimate the univariate LCSM, the following constraints were made: the latent change parameter was created by specifying the values at age 19 years as the indicator variable with a factor loading fixed to 1, the regression parameter between age 14 and age 19 years was fixed to 1 and the intercept and variance of age 19 were fixed to 0. This results in the latent change capturing the change between age 14 and 19 years and an estimate of the variance in the change factor. A regression parameter between age of 14 years and the change parameter was also included to investigate the degree to which change depends on baseline values (Kievit et al., 2018).

**Fig. 1:**
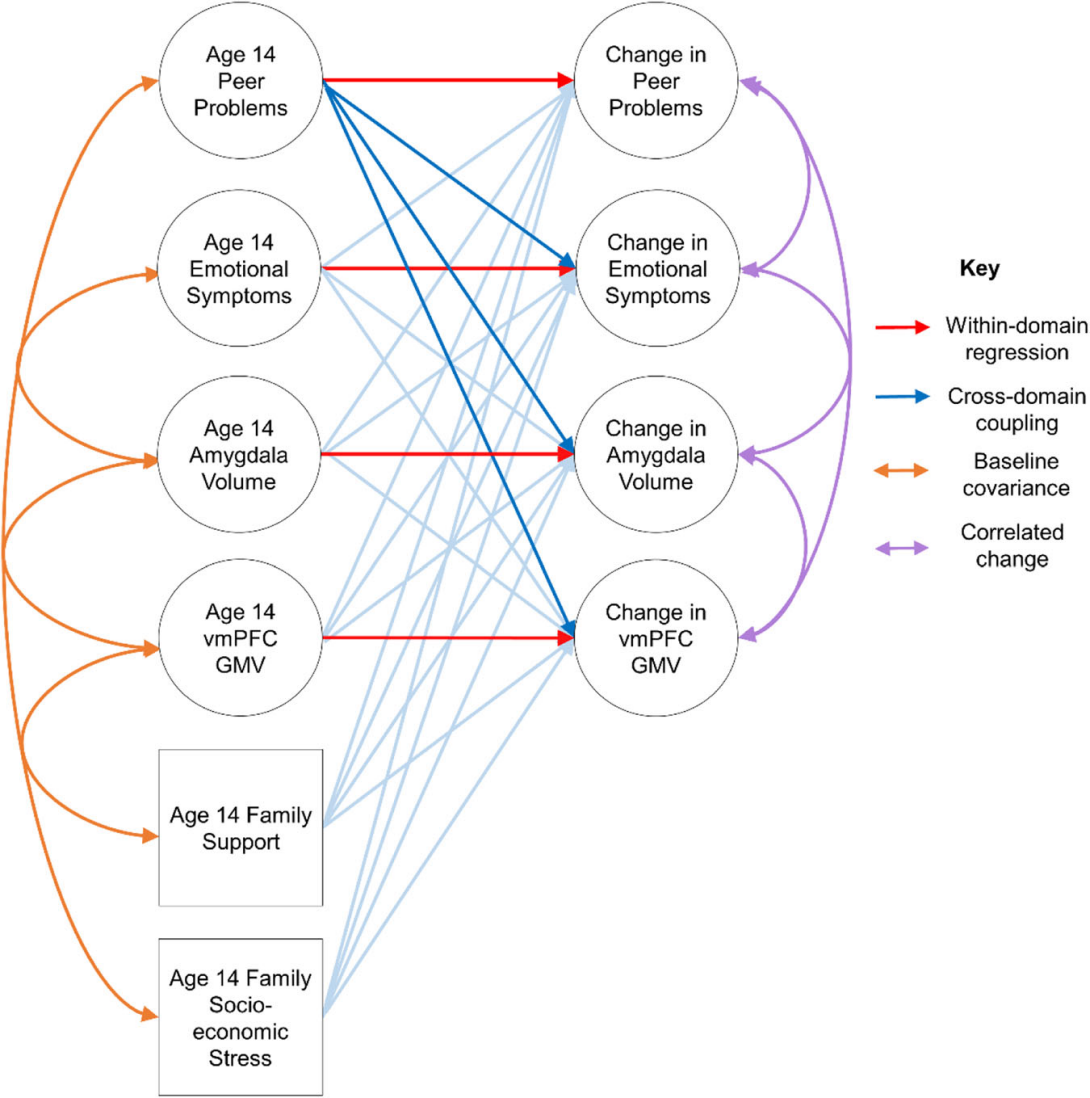
Multivariate latent change score models showing within-variable regression, cross-variable coupling, baseline covariance and correlated change between age 14 variables and change between ages 14 and 19 years. vmPFC ventromedial prefrontal cortex, GMV grey matter volume. Circles are latent variables and squares are observed variables. All possible parameters are included but the depiction is simplified for clarity

Next, to test the hypotheses, a bivariate model was run for peer problems and emotional symptoms, then separate trivariate models were run for peer problems, emotional symptoms and amygdala volume, and peer problems, emotional symptoms and vmPFC GMV separately. To see how these relationships change with the addition of other variables, a multivariate model was then constructed, with the hypothesized relationships between peer problems, emotional symptoms, amygdala volume and vmPFC GMV tested (see Fig. 1). The model was first tested without covariates, and then with the covariates specified previously.

#### Model Fit

Overall model fit was assessed by the robust chi-square (χ^2^) fit statistic, robust root mean squared error of approximation (RMSEA) with 90% confidence interval and robust comparative fit index (CFI). Rules of thumb were used to assess model fit. Good model fit was defined as robust χ^2^ *p*-value > 0.05, robust RMSEA < 0.06 (CI 0.00–0.08) and robust CFI > 0.95 (Hu & Bentler, 1999). A significant χ^2^ p-value is common in models with large sample sizes, so less emphasis was placed on this statistic. Comparative model fit was assessed by comparing the fit of nested, adjacent models through changes in fit statistics (changes in CFI values ≥ −0.01 and RMSEA values of ≥ +0.015 indicate poorer fit; Chen et al., 2008) and the scaled robust chi-square difference test statistic (significant difference indicates significantly poorer fit between models). Acceptable latent variable loadings were defined as at least 0.5 (Hair et al., 2010).

## Results

### Descriptive Statistics

Descriptive statistics for the continuous measures are shown in Table 1. The average age for both sexes was 14.40 years at the age 14 wave, and 18.90 years at the age 19 wave. There were slightly more females (509; 53%) compared to males (448; 47%) in the sample (χ^2^ (1) = 3.89, *p* = 0.04). Compared to males, females had significantly larger mean PDS score at age 14 years and greater total number of negative life events both before age 14 years and between ages 14 and 19 years. Males had significantly larger volumes in all brain regions of interest at both age 14 and 19 years.

**Table 1:** Descriptive statistics for continuous variables of interest, split by age and sex (*n* = 957)

Time point	Variable	Males (*n* = 448)			Females (*n* = 509)			Sex Difference Test (F (1, 956))
		M (SD)	Min	Max	M (SD)	Min	Max	
**Age 14 years**	Age at assessment (years)	14.40 (0.383)	13.20	15.44	14.40 (0.382)	13.26	15.45	F = 0.006
	Mean PDS score	2.6 (0.541)	1	4	3.2 (0.413)	1.6	4	F = 421.049***
	Family support	6.8 (1.513)	0	8	7 (1.325)	0	8	F = 5.340
	Family socioeconomic stress	0.6 (0.980)	0	6	0.5 (0.916)	0	5	F = 1.482
	LEQ total before age 14	5 (2.465)	0	13	5.5 (2.738)	0	16	F = 10.010**
	Whole brain volume (mm^3^)	1231347 (110543.553)	672637	1562514	1110175.6 (99191.488)	648827	1468714	F = 34.104***
	Left amygdala volume (mm^3^)	1803.4 (228.592)	947	2708.6	1624.6 (227.235)	998.3	2386.1	F = 0.925***
	Right amygdala volume (mm^3^)	1947 (269.733)	1018	3023.5	1756.1 (249.246)	902.3	2692.1	F = 146.764***
	Left vmPFC GMV (mm^3^)	5816.8 (792.657)	3514	8001	5294.4 (781.009)	2987	8093	F = 129.434***
	Right vmPFC GMV (mm^3^)	6028.4 (794.713)	2880	8034	5549.7 (744.618)	2603	7635	F = 105.094***
**Age 19 years**	Age at assessment (years)	18.90 (0.677)	17.80	21.20	18.90 (0.699)	17.90	22.44	F = 0.712
	LEQ total age 14–19	4.4 (2.916)	0	19	5.5 (3.096)	0	17	F = 92.425***
	Total childhood trauma score	6.6 (6.749)	0	48	6.1 (7.718)	0	64	F = 319.401
	Left amygdala volume (mm^3^)	1888.908 (228.476)	1298.4	2695.3	1677.5 (216.830)	1125.3	2729.4	F = 215.425***
	Right amygdala volume (mm^3^)	2025.356 (242.201)	1226	2804.6	1797.6 (237.788)	1181.7	2613.4	F = 214.832***
	Left vmPFC GMV (mm^3^)	5519.324 (759.670)	3207	8011	4927.8 (668.496)	3349	7290	F = 164.188***
	Right vmPFC GMV (mm^3^)	5729.364 (680.921)	4028	7769	5245.7 (647.921)	3410	7554	F = 126.610***

*PDS* Pubertal Development Score, *LEQ* Life Events Questionnaire, *vmPFC* ventromedial prefrontal cortex, *GMV* grey matter volume

Statistically significant difference between males and females:

**p < 0.01; ***p < 0.001

Ordinal and categorical descriptive statistics are shown in Online Resource 3. Recruitment site was not evenly split (χ^2^ (7) = 41.813, *p* < 0.001); more participants were recruited from places such as Dresden and Nottingham, however there was no difference in the recruitment center split between sex. Around 10% of the sample had a psychiatric diagnosis, with again no significant difference between sex. The distribution of psychiatric diagnoses in the sample, split by sex, is presented in Online Resource 4. Females were more likely to affirm greater emotional symptoms on all items at both ages 14 and 19. At age 14 years, peer problems responses were similar for most items between sex, except females were more likely to respond *‘Certainly true’* to *‘I have one good friend or more’* (χ^2^ (1) = 2.69, *p* = 0.04). At age 19 years, *‘I get along better with older people than with people my own age’* was different between sex, with females less likely to respond with *‘Not true’* compared to males (χ^2^ (1) = 3.35, *p* = 0.005).

### Measurement Invariance

#### Peer problems

Full loading invariance was demonstrated, however partial intercept invariance and partial residual invariance were achieved. Four intercepts and five residual variance parameters were freely estimated in the model. Overall measurement invariance model fit and comparative model fit is shown in Online Resource 5.

Four item intercepts were freely estimated in the model: ‘*Other people generally like me*’ for males and females, which had a greater mean at age 19 years compared to age 14 years and equality between sex, indicating that both males and females were equally more likely to affirm this item with older age. ‘*Other people pick on me or bully me*’ for males at age 19 years had a smaller item mean compared to other groups, indicating that this group was less likely to affirm this item. Furthermore, ‘*I get along better with older people than with people of my own age*’ at age 19 years for females had a greater item mean compared to other groups, again indicating greater likelihood of affirmation. Freeing these parameters resulted in good model fit; the mean differences of the latent variables can be compared, as specific item mean invariance was accounted for.

The residual variance for five parameters were freed: age 19 ‘*I would rather be alone than with other people*’ for males and females (with equality between sex), age 19 ‘*I have at least one good friend*’ for males, age 19 ‘*Other people pick on me or bully me*’ for females and age 19 ‘*I get along better with older people than with people of my own age*’ for males. The residual variance was <1 for these parameters, which shows that these items are more closely related to the latent variable ‘peer problems’ at age 19 compared to age 14. Online Resource 6 contains the item loadings from the strict invariance confirmatory factor analysis models.

#### Emotional Symptoms

Full loading invariance was achieved, and partial intercept invariance was established (see Online Resource 6 for full output). The item intercepts for ‘*I get a lot of headaches, stomach-aches or sickness*’ and ‘*I have many fears, I am easily scared*’ were smaller for age 19 males, compared to age 14 males/females and age 19 females. Furthermore, the intercept for ‘*I worry a lot*’ was larger for age 19 females compared to age 19 males and age 14 males/females. Full residual invariance was established so no additional constraints were made to the residual variance parameters.

### Univariate Latent Change Score Models

Full output from the results of the univariate latent change score models is shown in Table 2.

**Table 2:** Univariate latent change score model parameters for peer problems, emotional symptoms, amygdala volume and vmPFC GMV (*n* = 957)

Univariate latent change score models	Males (*n* = 448)				Females (*n* = 509)				Comparative model fit - Sex differences				
	Est	SE	Std.all	*p*	Est	SE	Std.all	*p*	χ^2^	df	*p*	ΔCFI	Δ RMSEA
**Peer problems**													
Mean at age 14 years	−0.135	0.044	−0.199	0.002	−0.202	0.041	−0.328	<0.001	1.188	1	0.276	0	0
Variance at age 14 years	0.458	0.064	1.000	<0.001	0.378	0.054	1.000	<0.001	0.907	1	0.341	+0.001	0
Mean change 14–19 years	0.277	0.061	0.448	<0.001	0.209	0.063	0.347	0.001	0.614	1	0.433	0	−0.001
Proportional covariance	−0.280	0.062	−0.668	<0.001	−0.261	0.050	−0.705	<0.001	0.089	1	0.766	+0.003	−0.001
Change variance	0.382	0.078	1.000	<0.001	0.362	0.060	1.000	<0.001	0.088	1	0.767	+0.003	−0.001
**Emotional symptoms**													
Mean at age 14 years	−0.599	0.054	−0.646	<0.001	0.201	0.046	0.228	<0.001***	109.69	1	<0.001	−0.104	+0.056
Variance at age 14 years	1.409	0.142	1.000	<0.001	0.772	0.085	1.000	<0.001	0.409	1	0.522	+0.001	−0.001
Mean change 14–19 years	0.139	0.080	0.117	0.081	0.056	0.065	0.056	0.390	0.652	1	0.419	0	−0.001
Proportional covariance	−0.421	0.097	−0.383	<0.001	−0.190	0.073	−0.217	0.009	3.003	1	0.083	−0.001	+0.001
Change variance	0.860	0.098	1.000	<0.001	0.993	0.106	1.000	<0.001	3.334	1	0.068	−0.001	+0.001
**Amygdala volume**													
Mean at age 14 years	1.807	0.011	9.375	<0.001	1.627	0.010	9.007	<0.001***	146.67	1	<0.001	−0.090	+0.148
Variance at age 14 years	0.037	0.004	1.000	<0.001	0.033	0.003	1.000	<0.001	0.815	1	0.367	0	−0.003
Mean change 14–19 years	0.081	0.009	0.589	<0.001	0.047	0.007	0.409	<0.001**	10.639	1	0.001	−0.006	+0.025
Proportional covariance	−0.013	0.002	−0.472	<0.001	−0.009	0.002	−0.451	<0.001	1.164	1	0.281	−0.001	0
Change variance	0.019	0.002	1.000	<0.001	0.013	0.003	1.000	<0.001*	4.063	1	0.043	−0.003	+0.011
**WBV-corrected amygdala volume**													
Mean at age 14 years	0.831	0.118	4.523	<0.001	0.393	0.088	2.314	<0.001**	8.884	1	0.002	−0.005	+0.012
Variance at age 14 years	0.026	0.003	0.776	<0.001	0.017	0.002	0.573	<0.001***	11.905	1	<0.001	−0.006	+0.013
Mean change 14–19 years	0.290	0.100	2.242	0.004	0.262	0.088	2.314	<0.001	0.839	1	0.839	0	−0.003
Proportional covariance	−0.010	0.002	−0.482	<0.001	−0.006	0.001	−0.440	<0.001*	4.257	1	0.039	−0.003	+0.003
Change variance	0.016	0.002	0.978	<0.001	0.011	0.002	0.966	<0.001*	4.798	1	0.029	−0.002	+0.005
Age 14 Amygdala volume ~ Age 14 WBV	0.788	0.095	0.473	<0.001	1.120	0.078	0.654	<0.001**	7.265	1	0.007	−0.004	+0.010
ΔAmygdala volume ~ Age 14 WBV	−0.174	0.081	−0.149	0.031	−0.198	0.082	−0.184	0.017	0.041	1	0.840	+0.001	−0.003
**vmPFC GMV**													
Mean at age 14 years	5.819	0.038	9.018	<0.001	5.293	0.033	8.531	<0.001***	116.22	1	<0.001	−0.079	+0.109
Variance at age 14 years	0.416	0.043	1.000	<0.001	0.385	0.041	1.000	<0.001	0.343	1	0.558	+0.001	−0.004
Mean change 14–19 years	−0.311	0.031	−0.585	<0.001	−0.356	0.025	−0.938	<0.001	1.358	1	0.244	0	−0.001
Proportional covariance	−0.188	0.031	−0.548	<0.001	−0.145	0.028	−0.615	<0.001	1.096	1	0.295	0	−0.001
Change variance	0.282	0.040	1.000	<0.001	0.144	0.029	1.000	<0.001**	8.926	1	0.003	−0.010	+0.022
**WBV-corrected vmPFC GMV**													
Mean at age 14 years	0.190	0.241	0.303	0.431	−0.432	0.224	−0.720	0.054*	3.976	1	0.046	−0.001	+0.002
Variance at age 14 years	0.140	0.018	0.356	<0.001	0.099	0.016	0.275	<0.001	3.307	1	0.069	−0.001	+0.002
Mean change 14–19 years	2.259	0.383	4.411	<0.001	2.045	0.314	5.582	<0.001	0.183	1	0.669	+0.001	−0.003
Proportional covariance	−0.064	0.016	−0.372	<0.001	−0.026	0.012	−0.279	0.027*	3.952	1	0.047	−0.001	+0.001
Change variance	0.209	0.029	0.799	<0.001	0.089	0.020	0.662	<0.001***	14.251	1	<0.001	−0.008	+0.016
Age 14 vmPFC GMV ~ Age 14 WBV	4.560	0.198	0.803	<0.001	5.159	0.203	0.852	<0.001*	5.064	1	0.024	−0.001	+0.002
ΔvmPFC GMV ~ Age 14 WBV	−2.082	0.309	−0.449	<0.001	−0.026	0.012	−0.279	0.027	0.027	1	0.870	+0.001	−0.004

*Est* estimate, *SE* standard error, *Std.all* standardized estimate (both latent and observed variables are standardized to have a variance of 1), *CFI* comparative fit index, *RMSEA* root mean square error of approximation, *vmPFC GMV* ventromedial prefrontal cortex grey matter volume, ~=predicted by

Statistically significant difference between males and females:

*p < 0.05; **p < 0.01; ***p < 0.001

#### Amygdala volume

For model identification, the first loading for the age 14 and 19 amygdala latent variables (left amygdala) was set to 1 and the intercepts set to 0. As the data were continuous, the robust maximum likelihood estimator was used. The model for amygdala volume was an excellent fit to the data (Robust χ^2^ (10) = 12.530, *p* = 0.251; CFI = 0.999; RMSEA = 0.023, 90% CI = [0.001, 0.058]). Amygdala volume at age 14 years was larger for males compared to females. There was significant individual variance in volume at age 14 years and the degree of variance was similar between sex. There was a mean-level increase in amygdala volume for both males and females, with evidence that males gained more than females. There were significant individual differences in change scores for both sexes, with some evidence that males showed greater change variance than females. As for proportional covariance, amygdala volume at age 14 years was negatively associated with change in volume between ages 14 and 19 years, with no significant difference between sex.

The amygdala model was run again with the addition of WBV as a predictor of amygdala volume at age 14 years and latent change in amygdala volume. This was to determine whether sex differences were due to differences in WBV. The WBV-corrected amygdala model was a good fit to the data (Robust χ^2^ (14) = 30.264, *p* = 0.007; CFI = 0.992; RMSEA = 0.049, 90% CI = [0.025, 0.074]). This model found that there was a significant difference in the chi-square test between males and females in amygdala volume mean and variance at age 14 years. However, the changes in CFI and RMSEA between models did not reach the standard cut-off to conclusively show sex differences. This was also the case for the sex differences in the proportional covariance, change variance, and the degree to which amygdala volume was predicted by WBV.

#### Ventromedial prefrontal cortex grey matter volume

Model specification was the same as with the amygdala model. The model for vmPFC GMV was a good fit to the data (Robust χ^2^ (10) = 20.307, *p* = 0.026; CFI = 0.993; RMSEA = 0.046, 90% CI = [0.015, 0.075]). Mean vmPFC GMV at age 14 years was larger for males compared to females; there was significant individual variance, but this was comparable between sex. There was a mean-level group decrease in vmPFC GMV for both males and females, with no difference between sex. There were significant individual differences in change scores for both sexes, with greater change variance for males compared to females. vmPFC volume at age 14 years was negatively associated with change in volume between ages 14 and 19 years, with no difference between sex.

As with the amygdala model, the vmPFC model was run again with the addition of WBV; this model was a good fit to the data (Robust χ^2^ (14) = 38.440, *p* < 0.001; CFI = 0.988; RMSEA = 0.061, 90% CI = [0.039, 0.085]). There was evidence that males had significantly greater change variance compared to females. Additional parameters had significant chi-square test statistics, but no significant differences in changes in CFI and RMSEA: age 14 vmPFC GMV, proportional covariance, and the degree to which WBV predicted vmPFC GMV at age 14 years.

### Multivariate Latent Change Score Models

A series of increasingly complex multivariate latent change score models were tested: first a bivariate model for peer problems and emotional symptoms, then trivariate models, a quadvariate model, and finally a covariate-corrected quadvariate model. Measurement invariance constraints identified previously were included in all models. The significant parameters of interest for all models are shown in Table 3.

**Table 3:** Multivariate latent change score models with statistically significant parameters of interest (*n* = 957)

	Males (*n* = 448)				Females (*n* = 509)			
Multivariate latent change score models	Est	SE	Std.all	*p*	Est	SE	Std.all	*p*
Bivariate model – PP and ES								
Correlated change: ΔPP and ΔES	**0.286**	**0.042**	**0.705**	**<0.001**	**0.251**	**0.032**	**0.762**	**<0.001**
Trivariate model – PP, ES, Amygdala								
Coupling: Age 14 Amyg vol → ΔPP	**0.471**	**0.239**	**0.134**	**0.048**	0.004	0.215	0.001	0.984
Correlated change: ΔPP and ΔES	**0.270**	**0.042**	**0.699**	**<0.001**	**0.245**	**0.034**	**0.762**	**<0.001**
Trivariate model – PP, ES, vmPFC								
Coupling: Age 14 PP → ΔvmPFC GMV	0.094	0.058	0.136	0.104	**0.130**	**0.059**	**0.259**	**0.027**
Correlated change: ΔPP and ΔES	**0.345**	**0.051**	**0.705**	**<0.001**	**0.306**	**0.040**	**0.765**	**<0.001**
Correlated change: ΔPP and ΔvmPFC GMV	**0.045**	**0.022**	**0.180**	**0.038**	−0.021	0.017	−0.150	0.213
Correlated change: ΔES and ΔvmPFC GMV	**0.062**	**0.028**	**0.155**	**0.024**	0.017	0.020	0.075	0.409
Quadvariate model – PP, ES, Amygdala, vmPFC								
Coupling: Age 14 PP → ΔvmPFC GMV	0.124	0.074	0.140	0.096	**0.169**	**0.077**	**0.261**	**0.028**
Correlated change: ΔPP and ΔES	**0.256**	**0.038**	**0.701**	**<0.001**	**0.232**	**0.030**	**0.765**	**<0.001**
Correlated change: ΔAmyg vol and ΔvmPFC GMV	**0.021**	**0.006**	**0.325**	**<0.001**	**0.014**	**0.003**	**0.404**	**<0.001**
Correlated change: ΔPP and ΔvmPFC GMV	**0.034**	**0.017**	**0.176**	**0.043**	−0.018	0.014	−0.163	0.177
Correlated change: ΔES and ΔvmPFC GMV	**0.058**	**0.026**	**0.151**	**0.028**	0.016	0.003	0.075	0.410
Covariate-corrected quadvariate model								
Coupling: Age 14 ES → ΔvmPFC GMV	−0.032	0.053	−0.045	0.551	−**0.089**	**0.044**	−**0.167**	**0.044**
Coupling: Age 14 Family support → ΔAmyg vol	−**0.017**	**0.007**	−**0.138**	**0.023**	0.004	0.005	0.040	0.421
Correlated change: ΔPP and ΔES	**0.171**	**0.033**	**0.623**	**<0.001**	**0.230**	**0.034**	**0.708**	**<0.001**
Correlated change: ΔAmyg vol and ΔvmPFC GMV	**0.026**	**0.004**	**0.497**	**<0.001**	**0.016**	**0.003**	**0.553**	**<0.001**
Correlated change: ΔPP and ΔvmPFC GMV	**0.026**	**0.010**	**0.215**	**0.011**	−0.017	0.012	−0.155	0.146
Correlated change: ΔES and ΔvmPFC GMV	**0.049**	**0.020**	**0.166**	**0.015**	0.014	0.016	0.077	0.378

Ages are in years. *PP* peer problems, *ES* emotional symptoms, *Amyg* *vol* amygdala volume, vmPFC *GMV* ventromedial prefrontal cortex grey matter volume, *Est* estimate, *SE* standard error, *Std.all* standardized estimate (both latent and observed variables are standardized to have a variance of 1), Δ = change in. Bold: statistically significant at the *p* < 0.05 level

#### Bivariate model

A bivariate latent change score was tested to investigate the relationship between peer problems and emotional symptoms between age 14 and 19 years. This model was a good fit to the data χ^2^ (352) = 464.125, *p* < 0.001; CFI = 0.969; RMSEA = 0.026, 90% CI = [0.019, 0.032].

**Fig. 2:**
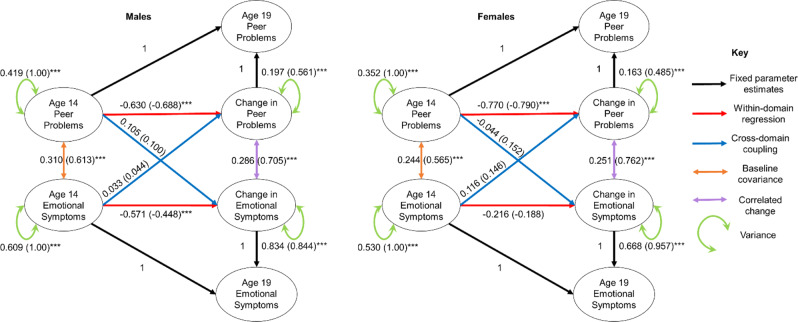
Bivariate latent change score model for peer problems and emotional symptoms. Indicator variables and mean structure have been omitted for clarity. Std.all parameters are presented in parentheses (standardized estimates; both latent and observed variables standardized to have a variance of 1). Statistical significance: *p < 0.05; **p < 0.01; ***p < 0.001

Parameter estimates are depicted in Fig. 2. For both males and females, the cross-domain coupling parameters were non-significant, indicating that there was no evidence that peer problems at age 14 years predicted change in emotional symptoms, or vice versa. However, even after accounting for significant baseline covariance, there was evidence for correlated change between peer problems and emotional symptoms (males std.all = 0.705, *p* < 0.001; females std.all = 0.762, *p* < 0.001). Fixing the correlated change parameter to equality between sex did not result in significantly worse fit, indicating no difference in the degree of correlated change between sex (χ^2^ (1) = 1.810, *p* = 0.179).

Discussion of the trivariate models of peer problems, emotional symptoms and amygdala volume and peer problems, emotional symptoms and vmPFC GMV are presented in Online Resource 8. Significant model parameters of interest are shown in Table 3.

#### Quadvariate model

The quadvariate model that included peer problems, emotional symptoms, amygdala volume and vmPFC GMV was a good fit to the data (χ^2^ (680) = 858.009, p = 0.001; CFI = 0.964; RMSEA = 0.023, 90% CI = [0.018, 0.028]). The only significant coupling parameter in this model was that peer problems at age 14 years predicted change in vmPFC GMV for females only (std.all = 0.261, *p* = 0.028). Amygdala volume did not predict change in peer problems as in the trivariate model. Correlated change was evident for peer problems and emotional symptoms (males std.all = 0.701, *p* < 0.001; females std.all = 0.765, *p* < 0.001), and amygdala volume and vmPFC GMV (males std.all = 0.325, *p* < 0.001; females std.all = 0.404, *p* < 0.001). For males only, there was also correlated change with peer problems and vmPFC GMV (std.all = 0.176, *p* = 0.043) and correlated change with emotional symptoms and vmPFC GMV (std.all = 0.151, *p* = 0.028).

#### Covariate-corrected quadvariate model

The additional covariates of interest of family support and family socioeconomic stress at age 14 years were included in this model, as well as recruitment center, mean PDS score at age 14 years, whether they had had a psychiatric diagnosis at age 14 years, and total number of negative life events before age 14 years. Total childhood trauma score and total negative life events between ages 14 and 19 years were also included. All covariates apart from total negative life events 14–19 years predicted the latent variables of interest at age 14 years; all covariates predicted the latent variables for change scores.

**Fig. 3:**
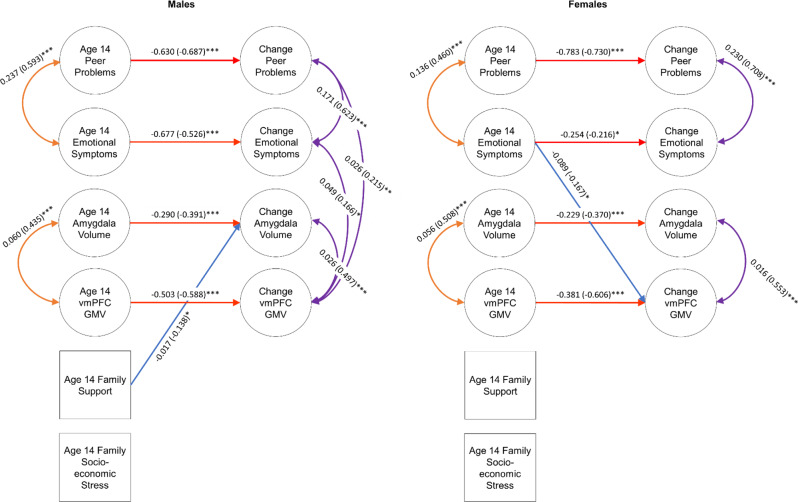
Significant paths for the covariate-corrected quadvariate latent change score model, separate for males and females

This model was an acceptable fit to the data (χ^2^ (1248) = 1614.117, *p* < 0.001; CFI = 0.923; RMSEA = 0.025, 90% CI = [0.021, 0.028]). The sub-optimal CFI value may have been due to the large number of additional parameters estimated in the model that were not statistically significant and therefore did not improve the model fit to the data. Peer problems at age 14 years were no longer a significant predictor of change in vmPFC GMV for females. However, in this model, age 14 emotional symptoms predicted change in vmPFC GMV for females (std.all = −0.167, *p* = 0.044). Greater emotional symptoms at age 14 years predicted a larger decrease in vmPFC GMV between ages 14 and 19 years for females. Stepwise addition of the covariates into the model revealed that this was driven by the addition of both total negative life events variables. Correlated change was evident for peer problems and emotional symptoms (males std.all = 0.623, *p* < 0.001; females std.all = 0.708, *p* < 0.001), and amygdala volume and vmPFC GMV (males std.all = 0.497, *p* < 0.001; females std.all = 0.553, *p* < 0.001). For males only, there was correlated change between the vmPFC GMV and peer problems (std.all = 0.215, *p* = 0.011), and between the vmPFC and emotional symptoms (std.all = 0.166, *p* = 0.015). To add, family support at age 14 years was found to predict change in amygdala volume in males (std.all = −0.138, *p* = 0.023), but not females (std.all = 0.040, *p* = 0.421). Males with greater family support score at age 14 years had a smaller increase in amygdala volume over time compared to those with lower family support. Significant parameters of interest are shown in Table 3, with the covariate-corrected quadvariate parameters specifically shown in Fig. 3. Regression and covariance output for the covariate-corrected quadvariate model is shown in Online Resource 9.

#### Sensitivity analyses

The relationship between family support at age 14 years and change in amygdala volume for males was verified in a separate analysis, which modelled family support as a latent variable. Full measurement invariance was achieved for the family support latent variable (see Online Resource 10) and the latent change score model for family support as a latent variable and amygdala volume is described in Online Resource 11.

Due to convergence issues with inclusion in the full covariate-corrected model, a separate model included WBV at age 14 years as a covariate of amygdala volume and vmPFC GMV at age 14 years and change between age 14 and 19 years, along with covariates of interest of family support and socioeconomic stress. Model fit was acceptable, although the CFI value was below the pre-defined cut-off of 0.95 (χ^2^ (808) = 1188.588, *p* < 0.001; CFI = 0.919; RMSEA = 0.031, 90% CI = [0.028, 0.035]). WBV at age 14 years predicted both amygdala volume (males std.all = 0.464, *p* < 0.001; females std.all = 0.634, *p* < 0.001) and vmPFC GMV at age 14 years (males std.all = 0.798, *p* < 0.001; females std.all = 0.843, *p* < 0.001). WBV at age 14 years predicted change in amygdala volume for both males only (males std.all = 0.265, *p* = 0.013). With the addition of WBV at age 14 years as a covariate, family support at age 14 years remained a significant predictor of change in amygdala volume for males (std.all = −0.112, *p* = 0.031). There was still correlated change between peer problems and emotional symptoms (males std.all = 0.714, *p* < 0.001; females std.all = 0.725, *p* < 0.001) and between amygdala volume and vmPFC GMV (males std.all = 0.328, *p* < 0.001; females std.all = 0.421, *p* < 0.001). Again, for males only, there was correlated change between the vmPFC GMV and peer problems (std.all = 1.335, *p* < 0.001), and between the vmPFC GMV and emotional symptoms (std.all = 0.159, *p* = 0.033).

Psychiatric diagnosis was used as a generic covariate in the analysis. To assess whether the range of psychiatric diagnoses present in the sample influenced the findings, additional analyses were conducted. Table S10 presents the distribution of psychiatric diagnoses in the sample, split by sex. A separate model tested whether results were similar if only mood or anxiety disorders were controlled for, instead of any psychiatric diagnosis, given the focus on emotional symptoms in the current study. Significant model parameters are presented in Online Resource 12. Associations of interest were similar to the previous covariate-corrected quadvariate model, however, age 14 emotional symptoms were no longer a significant predictor of change in vmPFC GMV for females (std.all = −0.146, *p* = 0.084).

## Discussion

Untangling the synergistic changes in social, emotional, and neuroanatomical factors in adolescence has implications for vulnerability and resilience to mental health problems such as anxiety and depression. To this end, the longitudinal interplay between peer problems, emotional symptoms, amygdala volume, and vmPFC GMV was examined across adolescence. Family support and socioeconomic stress in early adolescence were also investigated as predictors of change in the above variables of interest. Peer problems and emotional symptoms changed together for both sexes, but it was not the case that one affected the other. There were sex-specific findings: for males only, the vmPFC GMV changed together with peer problems and emotional symptoms, and family support predicted change in amygdala volume. Socioeconomic stress was not a predictor of change in peer problems, emotional symptoms, or regional brain volume for either sex. Exploration of the findings showed that greater total negative life events and higher levels of emotional symptoms predicted change in vmPFC GMV for females. This shows that there may be sex-specific interventions to promote brain development that supports socioemotional functioning.

For the first hypothesis, a directional relationship was not observed between peer problems and emotional symptoms. Rather, there was baseline covariance between values at age 14 years and correlated change between variables between ages 14 and 19 years. The magnitude of the relationship was similar between the sexes, and the relationship persisted even with the addition of covariates in the multivariate model. Previous research has found longitudinal links between peer relationships and internalizing problems across childhood and adolescence (Shin et al. 2016; Siegel et al., 2009; van Harmelen et al., 2016). The current study differed in the use of LCSMs, which simultaneously modelled different types of directional and associative relationships and allowed for a more robust detection of change that accounted for measurement error. Thus, previous research may have found correlated changes between peer problems and emotional symptoms, rather than a direction of the relationship. Indeed, more recent research using LCSMs supports this view; friendship quality did not predict subsequent resilient functioning across adolescence, rather these concepts changed together over time (van Harmelen et al., 2021). Modelling peer problems and emotional symptoms as latent variables included a range of indicators related to peer integration and victimization, such as having at least one good friend or being bullied. Other studies independently investigated different aspects of peer relationship problems that may have contributed to the differing results (e.g. peer victimization only, Siegel et al., 2009). Latent variable modelling and measurement invariance tests accounted for measurement error and ensured that the same concept was measured between sex and over time. There was partial measurement invariance for both peer problems and emotional symptoms that resulted in adjustments to the model, so that the latent means could be meaningfully compared between groups. For example, the item related to being bullied was less likely to be affirmed by males at age 19 years compared to age 19 females and both sexes at age 14, but this was not related to the latent variable of “peer problems”. Therefore, previous associations in specific components of peer problems may have been due to sex or age differences. The current study suggests that the core concepts of peer problems and emotional symptoms change together across adolescence.

In terms of the second hypothesis, there were originally some sex-specific findings: peer problems predicted change in vmPFC GMV for females only, and age 14 amygdala volume predicted change in peer problems for males only. However, these effects were not statistically significant when other predictors and covariates were entered into the model. Instead, there were unexpected findings which were found from the bidirectional investigation of relationships inherent to latent change score models. Emotional symptoms at age 14 years predicted change in vmPFC GMV for females, which was driven by the addition of negative life events before and after 14 years into the model, which also both predicted changes in vmPFC GMV. However, the effect of emotional symptoms on changes in vmPFC GMV was not found to be statistically significant when mood or anxiety disorders were controlled for. This may have been because the range of emotional symptoms were restricted; those with a mood or anxiety disorder had higher levels of emotional symptoms, which attenuated the association between emotional symptoms and change in vmPFC GMV. Together, this shows that both the presence of subjective negative life events and emotional distress in early adolescence affect the developmental trajectory of the vmPFC, which appears to trump the impact of peer problems. Associations between early life stress and regional prefrontal volume, including the vmPFC, have been found previously, with some studies suggesting that brain volume mediates the relationship between early life stress and emotional distress (Gorka et al., 2014; Hanson et al., 2010). Indeed, there is evidence to suggest that sex differences in the role of stress on the developing brain may be due to the interaction between sex-specific hormones and stress hormones, particularly in the PFC due to its protracted development into adolescence and adulthood (Shaw et al., 2020). This study shows that targeting the experience of negative life events and emotional distress may be necessary to buffer against the deleterious impact on vmPFC structural development in females.

For the third hypothesis, amygdala and vmPFC GMV did not mediate the association between peer problems and emotional symptoms; prior discussed results did not warrant mediation analyses. Instead, in the covariate-corrected multivariate model, there was correlated change between peer problems, emotional symptoms and vmPFC GMV for males. As with the previous finding of correlated change between peer problems and emotional symptoms, this was significant after accounting for baseline levels, changes within-variable, and changes between variables. Results from the univariate analysis in the current study found that males had a greater variance in change in vmPFC GMV compared to females during this age range. GMV in frontal regions have also been found to peak later and increase less rapidly in males compared to females, and male brain structure has been found to change more during childhood and early adolescence compared to females (Kaczkurkin et al., 2019; Lenroot et al., 2007). Taken together, this correlated change between peer problems, emotional symptoms, and vmPFC GMV may reflect a period of concerted change between social relationships, mental health, and frontal brain regions for males during this period rather than a specific direction of effect.

The fourth hypothesis was partially supported; greater parent-reported family support at age 14 years predicted less amygdala volume increase in males only. This remained statistically significant after correcting for WBV and mean PDS score, and when family support was modelled separately as a latent variable. This is in line with previous research which found that higher frequency of positive maternal behaviour predicted attenuated growth in the right amygdala for males between the ages of 12 and 16 years (Whittle et al., 2014). That previous research only found a significant effect in the right amygdala, however, this study modelled amygdala volume as a latent variable using both left and right amygdala volume as indicators. This suggests that the findings were able to uncover the association between family support and whole amygdala volume when these variables were not obscured by measurement error. There is also previous evidence that the rate of growth of the amygdala is associated with psychopathology; experience of Axis-I DSM-IV psychopathology between early and mid-adolescence has been associated with faster growth of the left amygdala (Whittle et al., 2013). Thus, the attenuated growth of the amygdala through family support may show a protective effect against psychopathology. At age 14 years, boys may still be reorienting their social focus from family to peers, therefore it may be a time that is sensitive to the secure base of family support (Jenkins et al., 2002). Girls, on the other hand, may have already completed this social transition in early adolescence, and are further along in neural development, so the same associations are not found as for boys (Kaczkurkin et al., 2019; Rueger et al., 2008). Future research should look at whether the patterns observed for males in this study are revealed earlier for females, such as in late childhood.

For the fifth hypothesis, socioeconomic stress did not predict changes in peer problems, emotional symptoms, or amygdala and vmPFC GMV. This was surprising given that previous research has found that low socioeconomic status is associated with less family support (Devenish et al., 2017) and that greater adolescent social stress was associated with smaller decreases in vmPFC GMV (Tyborowska et al., 2018). The reason for these results may be that socioeconomic stress was parent-reported, and thus the level of stress that the adolescent feels from the socioeconomic circumstances is unknown. Future research should aim to discern whether the adolescent’s perspective predicts socioemotional and neuroanatomical outcomes.

### Strengths and Limitations

The current study used the IMAGEN dataset, which is a rich, longitudinal dataset that contains social, psychological and neurobiological measures in adolescence (Schumann et al., 2010). The large sample size strengthened the ability to detect robust findings (Kievit et al., 2018). Additionally, IMAGEN participants were recruited from multiple European countries, which increases the generalizability of the findings. However, it must be highlighted that only participants of European descent were recruited in IMAGEN, which brings into question whether the interplay between peer and family relationships, mental health and neuroanatomy is similar in people of different ethnic backgrounds. Future research should explore this in a more diverse sample.

This analysis employed techniques such as latent change score modelling, which allowed interrogation different parameters of change and the potential direction of effects (Kievit et al., 2018). Measurement invariance tests were conducted to assess whether the concepts of peer problems and emotional symptoms (both measured by the SDQ) are similar between sex and over time. This has implications for the interpretation of change over time such as whether there is true change or the result of measurement error. This was particularly important because, in the IMAGEN dataset, there were slight differences in the wording of the items for the SDQ versions used at age 14 years (11–17-year-old version) and at age 19 years (17+ years version), for example, 11–17 *‘Other people my age generally like me’* and 17+*‘Other people generally like me’*. Measurement invariance tests revealed that the 17+ version was more likely to be affirmed compared to the 11–17 version for both sexes. This brings into question whether this was due to a difference with this age group or whether it was due to the wording of the item, which is broader. In this analysis, the item intercept was adjusted due to noninvariance; previous research has found that not adjusting for non-invariance between groups produced significant bias in regression parameter estimates in SEM (Guenole & Brown, 2014). This would be a problem for other studies that would want to compare longitudinal scores from adolescent to adult sample. In addition, low coefficient omega values suggested sub-optimal reliability which was different between the different age versions of the SDQ used. This brings into question the suitability of the SDQ items for investigating the concepts of interest and highlights the need to use other measures to reinforce the findings. Previous research has indicated that the SDQ has good psychometric properties from a cross-sectional sample of child and adolescent versions (Goodman, 2001) and the psychometric properties between adolescent and adult versions have been found to be similar in a cross-sectional sample (Brann et al., 2018). However, there is limited research into the psychometric performance of the SDQ when measuring change from the adolescent and adult versions. This is important with the proliferation of multi-year population cohort studies that assess changes throughout adolescence and extending into adulthood.

The correlated change observed in the current study can occur due to methodological issues or it may signify the presence of a third variable (Kievit et al. 2018; Könen & Karbach, 2021). This study attempted to address this by controlling for baseline scores and by including covariates that may be a common source of variance in the variables (Könen & Karbach, 2021). The covariate-corrected multivariate model included covariates such as stressful life events and psychiatric diagnosis, which had a minimal impact on the magnitude of the correlated change relationship between peer problems and emotional symptoms. It is possible, however, that variables not included in the study may have contributed to the change in both peer problems and emotional symptoms. Future research could investigate whether other aspects related to peer relationships, such as peer social skills (Nilsen et al., 2013; Segrin, 2000), explain the correlated change between peer problems and emotional symptoms for both sexes, and vmPFC GMV for males, during this age range.

### Implications

The findings suggest that there is concerted change between peer problems and emotional symptoms for both sexes, and this extends to vmPFC GMV for males. Furthermore, greater negative life events and higher levels of emotional symptoms predicted change in vmPFC GMV for females; for males, family support predict change in amygdala volume. This shows that, whilst there are commonalities between sexes, there are differences that may inform on the timing and targets for intervention—enhancing family support for males and protecting against negative life events and emotional symptoms for females. For the findings related to correlated change, it suggests that there is not a simple directional relationship between these variables, rather they are changing in concert. Further research is needed to elucidate whether these changes are being driven by a third variable not included in this study. This would have implications for where to direct potential intervention studies. For example, a social intervention targeted at reducing peer problems and increasing peer integration may show an association with change in emotional symptoms and vmPFC development, but that may have been driven by other factors, such as improving social skills rather than peer integration specifically.

## Conclusion

Concerted social, emotional, and neuroanatomical change in adolescence has implications for the development of mental health problems. The directions of these relationships—whether social relationships affect emotional symptoms, and whether it is mediated by structural development of specific brain regions—have been suggested by previous research. This study investigated these factors together using latent change score modelling. Rather than there being a specific direction of an effect between peer problems and emotional symptoms, these variables changed together during adolescence for both sexes. Sex-specific findings were evident: family support predicted amygdala volume for males longitudinally, and vmPFC GMV, peer problems, and emotional symptoms also changed together. For females, the nature of the latent change score analysis resulted in an unexpected finding that, with the addition of negative life events in the model, emotional symptoms predicted vmPFC GMV longitudinally, which replaced the effect of peer problems. These findings have implications for sex-specific targets for intervention and opens avenues for untangling the role of structural brain development in social and emotional functioning in adolescence. Future research can build on this to further specify test potential directional findings, such as whether the correlated change observed is due to other social variables not considered in the current study.

## Supplementary information

Online Resource 1

## References

[CR1] Bai X, Jiang L, Zhang Q, Wu T, Wang S, Zeng X, Li Y, Zhang L, Li J, Zhao Y, Dai J (2021). Subjective family socioeconomic status and peer relationships: Mediating roles of self-esteem and perceived stress. Frontiers in Psychiatry.

[CR2] Bernstein, D. P., Fink, L., Handelsman, L., & Foote, J. (1994). Childhood Trauma Questionnaire (CTQ) [Database record]. *APA PsycTests*. 10.1037/t02080-000

[CR3] Blakemore S-J (2008). The social brain in adolescence. Nature Reviews Neuroscience.

[CR4] Bora E, Fornito A, Pantelis C, Yücel M (2012). Gray matter abnormalities in Major Depressive Disorder: A meta-analysis of voxel-based morphometry studies. Journal of Affective Disorders.

[CR5] Brann P, Lethbridge MJ, Mildred H (2018). The young adult Strengths and Difficulties Questionnaire (SDQ) in routine clinical practice. Psychiatry Research.

[CR6] Brown, T. A. (2015). *Confirmatory Factor Analysis for Applied Research* (2nd ed.). Guilford Press Publications.

[CR7] Carskadon MA, Acebo C (1993). A self-administered rating scale for pubertal development. Journal of Adolescent Health.

[CR8] Chen F, Curran PJ, Bollen KA, Kirby J, Paxton P (2008). An empirical evaluation of the use of fixed cutoff points in RMSEA test statistic in Structural Equation Models. Sociological Methods & Research.

[CR9] Conger RD, Conger KJ (2002). Resilience in Midwestern families: Selected findings from the first decade of a prospective, longitudinal study. Journal of Marriage and Family.

[CR10] Conger RD, Donnellan MB (2007). An interactionist perspective on the socioeconomic context of human development. Annual Review of Psychology.

[CR11] Desikan RS, Ségonne F, Fischl B, Quinn BT, Dickerson BC, Blacker D, Buckner RL, Dale AM, Maguire RP, Hyman BT, Albert MS, Killiany RJ (2006). An automated labeling system for subdividing the human cerebral cortex on MRI scans into gyral-based regions of interest. NeuroImage.

[CR12] Devenish B, Hooley M, Mellor D (2017). The pathways between socioeconomic status and adolescent outcomes: A systematic review. American Journal of Community Psychology.

[CR13] Ducharme S, Albaugh MD, Hudziak JJ, Botteron KN, Nguyen T-V, Truong C, Evans AC, Karama S, Brain Development Cooperative Group. (2014). Anxious/depressed symptoms are linked to right ventromedial prefrontal cortical thickness maturation in healthy children and young adults. Cerebral Cortex.

[CR14] Essau CA, Olaya B, Anastassiou-Hadjicharalambous X, Pauli G, Gilvarry C, Bray D, O’callaghan J, Ollendick TH (2012). Psychometric properties of the Strength and Difficulties Questionnaire from five European countries. International Journal of Methods in Psychiatric Research.

[CR15] Fischl B, Salat DH, Busa E, Albert M, Dieterich M, Haselgrove C, van der Kouwe A, Killiany R, Kennedy D, Klaveness S, Montillo A, Makris N, Rosen B, Dale AM (2002). Whole brain segmentation. Neuron.

[CR16] Flora DB (2020). Your coefficient alpha is probably wrong, but which coefficient omega is right? A tutorial on using R to obtain better reliability estimates. Advances in Methods and Practices in Psychological Science.

[CR17] Giedd JN, Clasen LS, Lenroot R, Greenstein D, Wallace GL, Ordaz S, Molloy EA, Blumenthal JD, Tossell JW, Stayer C, Samango-Sprouse CA, Shen D, Davatzikos C, Merke D, Chrousos GP (2006). Puberty-related influences on brain development. Molecular and Cellular Endocrinology.

[CR18] Goddings A-L, Mills KL, Clasen LS, Giedd JN, Viner RM, Blakemore S-J (2014). The influence of puberty on subcortical brain development. NeuroImage.

[CR19] Goodman R (1997). The strengths and difficulties questionnaire: A research note. Journal of Child Psychology and Psychiatry.

[CR20] Goodman R (2001). Psychometric properties of the Strengths and Difficulties Questionnaire. Journal of the American Academy of Child & Adolescent Psychiatry.

[CR21] Goodman R, Ford T, Richards H, Gatward R, Meltzer H (2000). The Development and Well-Being Assessment: Description and initial validation of an integrated assessment of child and adolescent psychopathology. Journal of Child Psychology and Psychiatry.

[CR22] Goodman R, Yude C, Richards H, Taylor E (1996). Rating child psychiatric caseness from detailed case histories. Journal of Child Psychology and Psychiatry.

[CR23] Gorka, A. X., Hanson, J. L., Radtke, S. R., & Hariri, A. R. (2014). Reduced hippocampal and medial prefrontal gray matter mediate the association between reported childhood maltreatment and trait anxiety in adulthood and predict sensitivity to future life stress. *Biology of Mood & Anxiety Disorders*, *4*(12). 10.1186/2045-5380-4-12 10.1186/2045-5380-4-12 PMC4236295 25408863

[CR24] Guenole, N., & Brown, A. (2014). The consequences of ignoring measurement invariance for path coefficients in structural equation models. *Frontiers in Psychology*, *5*. 10.3389/fpsyg.2014.00980 10.3389/fpsyg.2014.00980 PMC4166111 25278911

[CR25] Hair JF, Anderson RE, Babin BJ, Black WC (2010). Multivariate data analysis: A global perspective (Vol. 7).

[CR26] Hanson JL, Chung MK, Avants BB, Shirtcliff EA, Gee JC, Davidson RJ, Pollak SD (2010). Early stress is associated with alterations in the orbitofrontal cortex: A tensor-based morphometry investigation of brain structure and behavioral risk. Journal of Neuroscience.

[CR27] Hiser J, Koenigs M (2018). The multifaceted role of the ventromedial prefrontal cortex in emotion, decision-making, social cognition, and psychopathology. Biological Psychiatry.

[CR28] Hu L, Bentler PM (1999). Cutoff criteria for fit indexes in covariance structure analysis: Conventional criteria versus new alternatives. Structural Equation Modeling.

[CR29] Huerta R, Brizuela-Gamiño O (2002). Interaction of pubertal status, mood and self-esteem in adolescent girls. The Journal of Reproductive Medicine.

[CR30] Jenkins SR, Goodness K, Buhrmester D (2002). Gender differences in early adolescents’ relationship qualities, self-efficacy, and depression symptoms. The Journal of Early Adolescence.

[CR31] Juchem C, Nixon TW, McIntyre S, Rothman DL, de Graaf RA (2010). Magnetic field homogenization of the human prefrontal cortex with a set of localized electrical coils. Magnetic Resonance in Medicine.

[CR32] Kaczkurkin AN, Raznahan A, Satterthwaite TD (2019). Sex differences in the developing brain: Insights from multimodal neuroimaging. Neuropsychopharmacology.

[CR33] Kaushik A, Kostaki E, Kyriakopoulos M (2016). The stigma of mental illness in children and adolescents: A systematic review. Psychiatry Research.

[CR34] Kelly PA, Viding E, Puetz VB, Palmer AL, Mechelli A, Pingault J-B, Samuel S, McCrory EJ (2015). Sex differences in socioemotional functioning, attentional bias, and gray matter volume in maltreated children: A multilevel investigation. Development and Psychopathology.

[CR35] Kievit RA, Brandmaier AM, Ziegler G, van Harmelen A-L, de Mooij SMM, Moutoussis M, Goodyer IM, Bullmore E, Jones PB, Fonagy P, Lindenberger U, Dolan RJ (2018). Developmental cognitive neuroscience using latent change score models: A tutorial and applications. Developmental Cognitive Neuroscience.

[CR36] Kim MJ, Loucks RA, Palmer AL, Brown AC, Solomon KM, Marchante AN, Whalen PJ (2011). The structural and functional connectivity of the amygdala: From normal emotion to pathological anxiety. Behavioural Brain Research.

[CR37] Kline, R. B. (2016). Coming of age. In R. B. Kline (Ed.), *Principles and practice of structural equation modeling* (4th ed., pp. 7–24). Guildford Press.

[CR38] Könen, T., & Karbach, J. (2021). Analyzing individual differences in intervention-related changes. *Advances in Methods and Practices in Psychological Science*, *4*(1). 10.1177/2515245920979172

[CR39] Lamblin M, Murawski C, Whittle S, Fornito A (2017). Social connectedness, mental health, and the adolescent brain. Neuroscience & Biobehavioral Reviews.

[CR40] Last A, Miles R, Wills L, Brownhill L, Ford T (2012). Reliability and sensitivity to change of the Family Life Questionnaire in a clinical population. Child and Adolescent Mental Health.

[CR41] Lenroot RK, Gogtay N, Greenstein DK, Wells EM, Wallace GL, Clasen LS, Blumenthal JD, Lerch J, Zijdenbos AP, Evans AC, Thompson PM, Giedd JN (2007). Sexual dimorphism of brain developmental trajectories during childhood and adolescence. NeuroImage.

[CR42] Lium, C. (2017). *Do parental internalizing symptoms and family stress predict child anxiety symptoms? Findings from a clinical trial* [Master thesis]. https://www.duo.uio.no/handle/10852/59711

[CR43] McArdle, J. J., & Hamagami, F. (2001). Latent difference score structural models for linear dynamic analyses with incomplete longitudinal data. In L. M. Collins & A. G. Sayer (Eds.), *New methods for the analysis of change*. (pp. 139–175). American Psychological Association. 10.1037/10409-005

[CR44] Mills KL, Goddings A-L, Clasen LS, Giedd JN, Blakemore S-J (2014). The developmental mismatch in structural brain maturation during adolescence. Developmental Neuroscience.

[CR45] Muris P, Meesters C, van den Berg S (2003). Internalizing and externalizing problems as correlates of self-reported attachment style and perceived parental rearing in normal adolescents. Journal of Child and Family Studies.

[CR46] Nelson EE, Jarcho JM, Guyer AE (2016). Social re-orientation and brain development: An expanded and updated view. Developmental Cognitive Neuroscience.

[CR47] Newcomb MD, Huba GJ, Bentler PM (1981). A multidimensional assessment of stressful life events among adolescents: Derivation and correlates. Journal of Health and Social Behavior.

[CR48] Nilsen W, Karevold E, Røysamb E, Gustavson K, Mathiesen KS (2013). Social skills and depressive symptoms across adolescence: Social support as a mediator in girls versus boys. Journal of Adolescence.

[CR49] Noonan MP, Mars RB, Sallet J, Dunbar RIM, Fellows LK (2018). The structural and functional brain networks that support human social networks. Behavioural Brain Research.

[CR50] Parr EJ, Shochet IM, Cockshaw WD, Kelly RL (2020). General belonging is a key predictor of adolescent depressive symptoms and partially mediates school belonging. School Mental Health.

[CR51] Powers A, Stevens JS, van Rooij SJH, Ely TD, Fani N, Jovanovic T, Ressler KJ, Bradley B (2017). Neural correlates and structural markers of emotion dysregulation in traumatized civilians. Social Cognitive and Affective Neuroscience.

[CR52] R Core Team. (2012). *R: A language and environment for statistical computing*. R Foundation for Statistical Computing.

[CR53] Rose AJ, Rudolph KD (2006). A review of sex differences in peer relationship processes: Potential trade-offs for the emotional and behavioral development of girls and boys. Psychological Bulletin.

[CR54] Rosseel Y (2012). lavaan: An R package for Structural Equation Modeling. Journal of Statistical Software.

[CR55] Rosseel, Y. (2022). *The lavaan tutorial*. https://lavaan.ugent.be/tutorial/tutorial.pdf

[CR56] Rothon C, Goodwin L, Stansfeld S (2012). Family social support, community “social capital” and adolescents’ mental health and educational outcomes: A longitudinal study in England. Social Psychiatry and Psychiatric Epidemiology.

[CR57] Rueger SY, Malecki CK, Demaray MK (2008). Gender differences in the relationship between perceived social support and student adjustment during early adolescence. School Psychology Quarterly.

[CR58] Rueger SY, Malecki CK, Pyun Y, Aycock C, Coyle S (2016). A meta-analytic review of the association between perceived social support and depression in childhood and adolescence. Psychological Bulletin.

[CR59] Schumann G, Loth E, Banaschewski T, Barbot A, Barker G, Büchel C, Conrod PJ, Dalley JW, Flor H, Gallinat J, Garavan H, Heinz A, Itterman B, Lathrop M, Mallik C, Mann K, Martinot J-L, Paus T, Poline J-B, Struve M (2010). The IMAGEN study: Reinforcement-related behaviour in normal brain function and psychopathology. Molecular Psychiatry.

[CR60] Segrin C (2000). Social skills deficits associated with depression. Clinical Psychology Review.

[CR61] Shaw GA, Dupree JL, Neigh GN (2020). Adolescent maturation of the prefrontal cortex: Role of stress and sex in shaping adult risk for compromise. Genes, Brain and Behavior.

[CR62] Shin KM, Cho S-M, Shin YM, Park KS (2016). Effects of early childhood peer relationships on adolescent mental health: A 6- to 8-year follow-up study in South Korea. Psychiatry Investigation.

[CR63] Siegel RS, La Greca AM, Harrison HM (2009). Peer victimization and social anxiety in adolescents: Prospective and reciprocal relationships. Journal of Youth and Adolescence.

[CR64] Tyborowska A, Volman I, Niermann HCM, Pouwels JL, Smeekens S, Cillessen AHN, Toni I, Roelofs K (2018). Early-life and pubertal stress differentially modulate grey matter development in human adolescents. Scientific Reports.

[CR65] van Harmelen A-L, Blakemore SJ, Goodyer IM, Kievit RA (2021). The interplay between adolescent friendship quality and resilient functioning following childhood and adolescent adversity. Adversity and Resilience Science.

[CR66] van Harmelen A-L, Gibson JL, Clair MCS, Owens M, Brodbeck J, Dunn V, Lewis G, Croudace T, Jones PB, Kievit RA, Goodyer IM (2016). Friendships and family support reduce subsequent depressive symptoms in at-risk adolescents. PLOS ONE.

[CR67] Van Roy B, Veenstra M, Clench-Aas J (2008). Construct validity of the five-factor Strengths and Difficulties Questionnaire (SDQ) in pre-, early, and late adolescence. Journal of Child Psychology and Psychiatry.

[CR68] Vijayakumar N, Allen NB, Dennison M, Byrne ML, Simmons JG, Whittle S (2017). Cortico-amygdalar maturational coupling is associated with depressive symptom trajectories during adolescence. NeuroImage.

[CR69] Wang J, Iannotti RJ, Luk JW, Nansel TR (2010). Co-occurrence of victimization from five subtypes of bullying: Physical, verbal, social exclusion, spreading rumors, and cyber. Journal of Pediatric Psychology.

[CR70] Whittle S, Dennison M, Vijayakumar N, Simmons JG, Yücel M, Lubman DI, Pantelis C, Allen NB (2013). Childhood maltreatment and psychopathology affect brain development during adolescence. Journal of the American Academy of Child & Adolescent Psychiatry.

[CR71] Whittle S, Simmons JG, Dennison M, Vijayakumar N, Schwartz O, Yap MBH, Sheeber L, Allen NB (2014). Positive parenting predicts the development of adolescent brain structure: A longitudinal study. Developmental Cognitive Neuroscience.

[CR72] Wierenga L, Langen M, Ambrosino S, van Dijk S, Oranje B, Durston S (2014). Typical development of basal ganglia, hippocampus, amygdala and cerebellum from age 7 to 24. NeuroImage.

[CR73] Wu H, Estabrook R (2016). Identification of Confirmatory Factor Analysis models of different levels of invariance for ordered categorical outcomes. Psychometrika.

[CR74] Yao S, Zhang C, Zhu X, Jing X, Mcwhinnie C, Abela J (2009). Measuring adolescent psychopathology: Psychometric properties of the self-report Strengths and Difficulties Questionnaire in a sample of Chinese adolescents. The Journal of Adolescent Health.

