# Supplementary material for: Longitudinal Associations between Peer and Family Relationships, Emotional Symptoms, and Regional Brain Volume across Adolescence
Source: J Youth Adolesc. 2023 Feb 20;52(4):734–53. doi: 10.1007/s10964-023-01740-7 (PMC9957881; doi:10.1007/s10964-023-01740-7)
Supplement: Supplementary file 1 — Online Resource 1 [file 10964_2023_1740_MOESM1_ESM.docx]

**Online Resource 1**

**Differences between the sample where data quality issues were removed and the complete case sample**

To investigate potential bias in the sample, the complete-case sample (CC sample, n = 957) was compared to the sample where data were removed due to quality issues identified by IMAGEN (DQ sample, n = 2302). Groups were compared by dummy coding participant presence in the CC sample within the DQ sample dataset.

First, two-sample t-tests were conducted to compare values between samples in the 15 continuous variables described in the main manuscript (see Table S1), separately for males and females. For males, the DQ sample had a greater family socioeconomic stress score at age 14 years, greater total negative life events before age 14 years and greater total childhood trauma score compared to the CC sample. However, these differences were not statistically significant when False Discovery Rate (FDR) correction was applied. For females, the DQ sample had lower family support score at age 14 years, and greater family socioeconomic stress score at age 14 years, greater total negative life events before age 14 years, greater total negative life events between ages 14 and 19 years and greater total childhood trauma compared to the CC sample. When applying the FDR-correction, family support, family socioeconomic stress and total childhood trauma score remained statistically significant.

**Table S1**

*Statistically significant t-test differences between the Data Quality (DQ) and Complete Case (CC) samples, with false discovery rate correction*

|  | **Variable** | **DQ Sample** | **CC sample** | **t-test** | **FDR-corrected**  **p-value (n = 15 tests per sex)** |
| --- | --- | --- | --- | --- | --- |
| **Males** |  | **n = 689** | **n = 448** |  |  |
|  |  | **M (SD)** | **M (SD)** |  |  |
|  | Family SE stress score at age 14 years | 0.79 (1.19) | 0.62 (0.98) | *t*(1049.10) = 2.545, *p* = 0.011 | *p* = 0.083 |
|  | LEQ negative total before age 14 years | 5.35 (2.65) | 4.99 (2.47) | *t*(997.24) = 2.278, *p* = 0.023 | *p* = 0.114 |
|  | Total childhood trauma score | 8.17 (7.75) | 6.57 (6.75) | *t*(353.22) = 2.56, *p* = 0.011 | *p* = 0.083 |
|  | Right vmPFC GMV at age 19 years | 5847.03 (771.82) | 5729.36 (680.92) | *t*(426.83) = 1.969, *p* = 0.049 | *p* = 0.186 |
| **Females** |  | **n = 668** | **n = 509** |  |  |
|  |  | **M (SD)** | **M (SD)** |  |  |
|  | Family support score at age 14 years | 10.75 (1.57) | 10.98 (1.33) | *t*(1111.9) = -2.683, *p* < 0.001 | *p* = 0.037 |
|  | Family SE stress at age 14 years | 0.89 (1.29) | 0.55 (0.92) | *t*(1083.7) = 5.156, *p* < 0.001 | *p* *<* 0.001 |
|  | LEQ negative total before age 14 years | 5.90 (2.83) | 5.52 (2.74) | *t*(1079.1) = 2.231, *p* = 0.026 | *p* = 0.078 |
|  | LEQ negative total age 14-19 years | 6.24 (3.43) | 5.55 (3.10) | *t*(241.51) = 2.271, *p* = 0.024 | *p* = 0.078 |
|  | Total childhood trauma score | 9.09 (11.34) | 6.12 (7.72) | *t*(285.09) = 3.456, *p* < 0.001 | *p* = 0.005 |

*Note:* CC = complete-case, DQ = data quality, FDR = false discovery rate, LEQ = Life Events Questionnaire, SE = socioeconomic

Chi-square tests were conducted to investigate differences between samples in ordinal and categorical level variables (22 variables, see Table S2).

For males, worries at age 19 years and recruitment centre distribution were different between samples after FDR correction. FDR-corrected post-hoc tests showed that, for worries at age 19 years, participants in the CC sample were more likely to answer “Not true” compared to the DQ sample (p < 0.001) whilst the DQ sample was more likely to answer “Somewhat true” compared to the CC sample (p = 0.023). For recruitment centre, in the DQ sample, a greater proportion of participants recruited from Berlin (p < 0.001) and Dublin (p = 0.028), whilst, for the CC sample, a greater proportion of participants was recruited from Dresden (p = 0.027) and Paris (0.041).

For females, only recruitment centre distribution was different between samples after FDR correction. FDR-corrected post-hoc tests showed that a greater proportion of participants were recruited from Dresden in the CC sample compared to the DQ sample (p = 0.002).

**Table S2**

*Statistically significant chi-square test differences between the Data Quality (DQ; males n = 689, females n = 668) and Complete Case (CC; males n = 448, females n = 509) samples, with false discovery rate correction*

|  | **Variable** | ***Χ^2^* test** | **FDR-corrected**  **p-value (n = 22 tests per sex)** |
| --- | --- | --- | --- |
| **Males** | Worries at age 19 years | *Χ^2^*(2) = 15.297, *p* < 0.001 | *p* < 0.001 |
|  | Bullied at age 19 years | *Χ^2^*(2) = 7.283, *p* = 0.026 | *p* = 0.144 |
|  | Psychiatric diagnosis at age 14 years* | *Χ^2^*(1) = 7.283, *p* = 0.026 | *p* = 0.144 |
|  | Recruitment centre | *Χ^2^*(7) = 45.159, *p* < 0.001 | *p* < 0.001 |
| **Females** | Worries at age 19 years | *Χ^2^*(2) = 8.441, *p* = 0.015 | *p* = 0.108 |
|  | Unhappy at age 19 years | *Χ^2^*(2) = 7.503, *p* = 0.023 | *p* = 0.129 |
|  | Old best at age 14 years | *Χ^2^*(2) = 6.706, *p* = 0.035 | *p* = 0.154 |
|  | Friend at age 19 years | *Χ^2^*(2) = 6.312, *p* = 0.042 | *p* = 0.156 |
|  | Old best at age 19 years | *Χ^2^*(2) = 8.665, *p* = 0.013 | *p* = 0.108 |
|  | Recruitment centre | *Χ^2^*(2) = 28.719, *p* < 0.001 | *p* = 0.004 |

*Note:* * = Yates’ continuity correction applied

**Online Resource 2**

*Questionnaire items for the UK versions peer problems and emotional symptoms scales at age 14 and 19 years. Short versions are shown in parentheses*

| **Questionnaire** | **SDQ 11-17 years Peer Problems** | **SDQ 17+ years Peer Problems** | **SDQ 11-17 years Emotional Symptoms** | **SDQ 17+ years Emotional Symptoms** |
| --- | --- | --- | --- | --- |
| **Age used** | 14 years | 19 years | 14 years | 19 years |
| **Items** | I am usually on my own. I generally play alone or keep to myself* (Loner) | I would rather be alone than with other people* (Loner) | I get a lot of headaches, stomach-aches or sickness (Somatic) | I get a lot of headaches, stomach-aches or sickness (Somatic) |
|  | I have one good friend or more* (Friend) | I have at least one good friend* (Friend) | I worry a lot (Worries) | I worry a lot (Worries) |
|  | Other people my age generally like me* (Popular) | Other people generally like me* (Popular) | I am often unhappy, down-hearted or tearful* (Unhappy) | I am often unhappy, depressed or tearful* (Unhappy) |
|  | Other children or young people pick on me or bully me* (Bully) | Other people pick on me or bully me* (Bully) | I am nervous in new situations. I easily lose confidence (Clingy) | I am nervous in new situations. I easily lose confidence (Clingy) |
|  | I get on better with adults than with people my own age* (Old best) | I get along better with older people than with people my own age* (Old best) | I have many fears, I am easily scared (Afraid) | I have many fears, I am easily scared (Afraid) |

*Note.* * = Differences in wording between versions

**Online Resource 3**

*Descriptive statistics for ordinal and categorical variables of interest, split by age and sex (n = 957)*

|  | | | **Males (n = 448)** | | | | | **Females (n = 509)** | | | |
| --- | --- | --- | --- | --- | --- | --- | --- | --- | --- | --- | --- |
|  | | | **Response Options n (%)** | | | | | **Response Options n (%)** | | | |
| **Age 14 years** |  | **Berlin** | | **Dresden** | **Dublin** | **Hamburg** | **Berlin** | | **Dresden** | **Dublin** | **Hamburg** |
|  | Recruitment Centre | 30 (6.7) | | 75 (16.7) | 38 (8.5) | 61 (13.6) | 50 (9.8) | | 77 (15.1) | 44 (8.6) | 61 (12.0) |
|  |  | **London** | | **Mannheim** | **Nottingham** | **Paris** | **London** | | **Mannheim** | **Nottingham** | **Paris** |
|  |  | 51 (11.4) | | 53 (11.8) | 71 (15.8) | 69 (15.4) | 77 (15.1) | | 60 (11.8) | 72 (14.1) | 68 (13.4) |
|  |  | **Yes** | | **No** |  |  | **Yes** | | **No** |  |  |
|  | Psychiatric Diagnosis | 41 (9.2%) | | 407 (90.8%) |  |  | 65 (12.8%) | | 444 (87.2%) |  |  |
|  |  | **Not true** | | **Somewhat true** | **Certainly true** |  | **Not true** | | **Somewhat true** | **Certainly true** |  |
|  | I get a lot of headaches, stomach-aches or sickness*** | 336 (75%) | | 92 (20.5%) | 20 (4.5%) |  | 273 (53.6%) | | 194 (38.1%) | 42 (8.3%) |  |
|  | I worry a lot*** | 221 (49.3%) | | 181 (40.4%) | 46 (10.3%) |  | 150 (29.5%) | | 255 (50.1%) | 104 (20.4%) |  |
|  | I am often unhappy, down-hearted or tearful*** | 370 (82.6%) | | 68 (15.2%) | 10 (2.2%) |  | 309 (60.7%) | | 171 (33.6%) | 29 (5.7%) |  |
|  | I am nervous in new situations. I easily lose confidence*** | 241 (53.8%) | | 161 (35.9%) | 46 (10.3%) |  | 175 (34.4%) | | 231 (45.4%) | 103 (20.2%) |  |
|  | I have many fears, I am easily scared*** | 349 (77.9%) | | 92 (20.5%) | 7 (1.6%) |  | 298 (58.5%) | | 181 (35.6%) | 30 (5.9%) |  |
|  | I am usually on my own. I generally play alone or keep to myself | 260 (58%) | | 150 (33.5%) | 38 (8.5%) |  | 298 (58.5%) | | 183 (36%) | 28 (5.5%) |  |
|  | I have one good friend or more (negative loading)* | 7 (1.6%) | | 43 (9.6%) | 398 (88.8%) |  | 4 (0.8%) | | 28 (5.5%) | 477 (93.7%) |  |
|  | Other people my age generally like me (negative loading) | 18 (4%) | | 208 (46.4%) | 222 (49.6%) |  | 23 (4.5%) | | 225 (44.2%) | 261 (51.3%) |  |
|  | Other children or young people pick on me or bully me | 373 (83.3%) | | 64 (14.3%) | 11 (2.5%) |  | 430 (84.5%) | | 63 (12.4%) | 16 (3.1%) |  |
|  | I get on better with adults than with people my own age** | 262 (58.5%) | | 153 (34.2%) | 33 (7.4%) |  | 306 (60.1%) | | 179 (35.2%) | 24 (4.7%) |  |
| **Age 19 years** |  | **Not true** | | **Somewhat true** | **Certainly true** |  | **Not true** | | **Somewhat true** | **Certainly true** |  |
|  | I get a lot of headaches, stomach-aches or sickness*** | 358 (79.9%) | | 76 (17%) | 14 (3.1%) |  | 274 (53.8%) | | 174 (34.2%) | 61 (12%) |  |
|  | I worry a lot*** | 208 (46.4%) | | 163 (36.4%) | 77 (17.2%) |  | 100 (19.6%) | | 252 (49.5%) | 157 (30.8%) |  |
|  | I am often unhappy, depressed or tearful*** | 348 (77.7%) | | 86 (19.2%) | 14 (3.1%) |  | 286 (56.2%) | | 174 (34.2%) | 49 (9.6%) |  |
|  | I am nervous in new situations. I easily lose confidence*** | 217 (48.4%) | | 181 (40.4%) | 50 (11.2%) |  | 177 (34.8%) | | 226 (44.4%) | 106 (20.8%) |  |
|  | I have many fears, I am easily scared*** | 386 (86.2%) | | 56 (12.5%) | 6 (1.3%) |  | 308 (60.5%) | | 162 (31.8%) | 39 (7.7%) |  |
|  | I would rather be alone than with other people | 221 (49.3%) | | 206 (46%) | 21 (4.7%) |  | 255 (50.1%) | | 234 (46%) | 20 (3.9%) |  |
|  | I have at least one good friend (negative loading) | 3 (0.7%) | | 27 (6.0%) | 418 (93.3%) |  | 3 (0.6%) | | 36 (7.1%) | 470 (92.3%) |  |
|  | Other people generally like me (negative loading) | 7 (1.6%) | | 146 (32.6%) | 295 (65.8%) |  | 5 (1.0%) | | 147 (28.9%) | 357 (70.1%) |  |
|  | Other people pick on me or bully me | 419 (93.5%) | | 26 (5.8%) | 3 (0.7%) |  | 467 (91.7%) | | 38 (7.5%) | 4 (0.8%) |  |
|  | I get along better with older people than with people my own age*** | 207 (46.2%) | | 189 (42.2%) | 52 (11.6%) |  | 181 (35.6%) | | 250 (49.1%) | 78 (15.3%) |  |

*Note.* Statistically significant difference between males and females: * p < 0.05; ** p < 0.01; *** p < 0.001.

**Online Resource 4**

*Distribution of psychiatric diagnoses (DSM-IV or ICD-10) in the sample, split by sex*

|  |  |  |  |
| --- | --- | --- | --- |
| Diagnosis (DSM-IV or ICD-10) | Male n | Female n | Total n |
| ADHD/Autism | 21 | 9 | 30 |
| Mood Disorder | 11 | 47 | 58 |
| Anxiety Disorder | 6 | 34 | 40 |
| Conduct/Oppositional Disorder | 9 | 8 | 17 |
| Other Disorder | 8 | 11 | 19 |
| **Total** | 55 | 109 | 164 |

*Note.* ADHD/Autism: ADHD Combined, ADHD Hyperactive, ADHD Impulsive, ADHD Other, ADHD Any, PDD/Autism; Mood Disorder: Emotional disorder, Major depression, Mania/Bipolar, Other depression; Anxiety Disorder: Agoraphobia, Generalised anxiety disorder, OCD, Other anxiety disorder, Panic disorder, PTSD, Separation anxiety, Social phobia, Specific phobia; Conduct/Oppositional Disorder: Any Conduct/Oppositional Disorder, Conduct disorder, Oppositional defiant disorder, Other disruptive disorder; Other Disorder: Other disorder, Eating disorder, Tic disorder. Some participants had more than one diagnosis, which is why the total is not equal to the psychiatric diagnosis variable.

**Online Resource 5**

*Measurement invariance models for the peer problems between sex and across time*

|  | **Robust model fit** | | |  |  |  | **Robust χ^2^  difference test** | | |  |
| --- | --- | --- | --- | --- | --- | --- | --- | --- | --- | --- |
| **Model** | χ^2^ | df | *p* | CFI | RMSEA  [90% CI] | χ^2^ | | df | *p* | |
| Configural Invariance | 73.162 | 58 | 0.087 | 0.981 | 0.023  [0.001-0.039] | - | | - | *-* | |
| Full Loading Invariance | 85.604 | 70 | 0.099 | 0.980 | 0.022  [0.001-0.036] | 12.817 | | 12 | 0.383 | |
| Full Intercept Invariance | 249.268 | 82 | <0.001 | 0.788 | 0.065  [0.056-0.073] | 133.59 | | 12 | <0.001 | |
| Partial Intercept Invariance | 99.962 | 79 | 0.056 | 0.973 | 0.024  [0.001-0.037] | 13.407 | | 9 | 0.145 | |
| Full Residual Invariance | 171.309 | 94 | <0.001 | 0.902 | 0.042  [0.031-0.051] | 61.525 | | 15 | <0.001 | |
| Partial Residual Invariance | 117.490 | 90 | 0.027 | 0.965 | 0.025  [0.009-0.037] | 16.707 | | 11 | 0.117 | |

**Online Resource 6**

*Item loadings for the individual strict invariance confirmatory factor analysis models, including estimate, standard error, standardised estimate (std.all) and p-value (N = 957; male n = 448, female n = 509)*

| **Latent Variable** | **Indicators** | **Estimate** | **SE** | **Male std.all** | ***p*** |  | **Female std.all** | ***p*** |
| --- | --- | --- | --- | --- | --- | --- | --- | --- |
| Age 14 Peer Problems | Loner | 0.583 | 0.067 | 0.504 | <0.001 |  | 0.468 | <0.001 |
|  | Friend | -0.920 | 0.126 | -0.677 | <0.001 |  | -0.641 | <0.001 |
|  | Popular | -0.805 | 0.084 | -0.627 | <0.001 |  | -0.590 | <0.001 |
|  | Bullied | 0.773 | 0.097 | 0.612 | <0.001 |  | 0.575 | <0.001 |
|  | Old Best | 0.626 | 0.073 | 0.531 | <0.001 |  | 0.494 | <0.001 |
| Age 19 Peer Problems | Loner |  |  | 0.500 | <0.001 |  | 0.453 | <0.001 |
|  | Friend |  |  | -0.763 | <0.001 |  | -0.536 | <0.001 |
|  | Popular |  |  | -0.534 | <0.001 |  | -0.486 | <0.001 |
|  | Bullied |  |  | 0.518 | <0.001 |  | 0.649 | <0.001 |
|  | Old Best |  |  | 0.386 | <0.001 |  | 0.397 | <0.001 |
| Age 14 Emotional Symptoms | Worries | 1.013 | 0.087 | 0.712 | <0.001 |  | 0.692 | <0.001 |
|  | Clingy | 0.642 | 0.056 | 0.540 | <0.001 |  | 0.520 | <0.001 |
|  | Somatic | 0.456 | 0.048 | 0.415 | <0.001 |  | 0.397 | <0.001 |
|  | Unhappy | 1.008 | 0.096 | 0.710 | <0.001 |  | 0.691 | <0.001 |
|  | Afraid | 0.764 | 0.071 | 0.607 | <0.001 |  | 0.586 | <0.001 |
| Age 19 Emotional Symptoms | Worries |  |  | 0.794 | <0.001 |  | 0.789 | <0.001 |
|  | Clingy |  |  | 0.637 | <0.001 |  | 0.631 | <0.001 |
|  | Somatic |  |  | 0.506 | <0.001 |  | 0.501 | <0.001 |
|  | Unhappy |  |  | 0.792 | <0.001 |  | 0.788 | <0.001 |
|  | Afraid |  |  | 0.701 | <0.001 |  | 0.696 | <0.001 |

Note. The estimate and SE are the same between sex and across time and so are only presented once.

**Online Resource 7**

*Measurement invariance models for the emotional symptoms latent variable between sex and across time*

|  | **Robust model fit** | | |  |  | **Robust χ^2^  difference test** | | |
| --- | --- | --- | --- | --- | --- | --- | --- | --- |
| **Model** | χ^2^ | df | *p* | CFI | RMSEA | χ^2^ | df | *p* |
| Configural Invariance | 81.849 | 58 | 0.021 | 0.990 | 0.029 [0.012-0.043] | - | - | *-* |
| Full Loading Invariance | 100.255 | 70 | 0.010 | 0.988 | 0.030  [0.015-0.043] | 16.973 | 12 | 0.151 |
| Full Intercept Invariance | 155.546 | 82 | <0.001 | 0.971 | 0.043  [0.033-0.054] | 44.412 | 12 | <0.001 |
| Partial Intercept Invariance | 109.448 | 79 | 0.013 | 0.988 | 0.028 [0.014-0.041] | 9.7638 | 9 | 0.370 |
| Full Residual Invariance | 133.471 | 94 | 0.005 | 0.984 | 0.030 [0.017-0.041] | 22.70 | 15 | 0.091 |

**Online Resource 8**

**Model fit and interpretation for the trivariate latent change score models**

***Peer Problems, Emotional Symptoms and Amygdala Volume – Trivariate Model***

This model was a good fit to the data (χ^2^ (506) = 650.154, p < .001; CFI = 0.966; RMSEA = 0.024, 90% CI = [0.018, 0.030]. Amygdala volume at age 14 years predicted change in peer problems for males only (std.all = 0.134, p = 0.048). In males with a low peer problems score at age 14 years, those with larger amygdala volume at age 14 years had a greater increase in peer problems score between age 14 and 19 years compared to those with a smaller amygdala volume at age 14 years. There was still correlated change between peer problems and emotional symptoms for males (std.all = 0.699, p < 0.001) and females (std.all = 0.762, p < 0.001). As there was no evidence that peer problems predicted either emotional symptoms or amygdala volume, mediation analysis was not conducted.

***Peer Problems, Emotional Symptoms and vmPFC GMV – Trivariate Model***

This model was a good fit to the data (χ^2^ (506) = 616.826, p = 0.001; CFI = 0.973; RMSEA = 0.021, 90% CI = [0.015, 0.027]. Peer problems at age 14 years predicted change in vmPFC GMV for females only (std.all = 0.259, p = 0.027). In females with larger vmPFC GMV at age 14 years, those with greater peer problems score at age 14 years showed smaller loss of vmPFC GMV compared to those with lower peer problems score at age 14 years. There was still correlated change between peer problems and emotional symptoms for males (std.all = 0.705, p < 0.001) and females (std.all = 0.765, p < 0.001). There was also correlated change between vmPFC GMV and peer problems for males only (std.all = 0.180, p = 0.038), and vmPFC GMV and emotional symptoms also showed correlated change for males only (std.all = 0.155, p = 0.024).

**Online Resource 9**

*Regression and covariance output for the covariate-corrected quadvariate model (n = 957)*

|  | **Males (n = 448)** | | | | **Females (n = 509)** | | | |
| --- | --- | --- | --- | --- | --- | --- | --- | --- |
| **Parameters** | Est | SE | Std.all | *p* | Est | SE | Std.all | *p* |
| Age 14 ES ~ |  |  |  |  |  |  |  |  |
| Age 14 SE Stress | -0.056 | 0.048 | -0.072 | 0.239 | -0.001 | 0.043 | -0.002 | 0.977 |
| Age 14 Fam Supp | -0.009 | 0.032 | -0.018 | 0.774 | -0.024 | 0.029 | -0.044 | 0.402 |
| **Recruit Dresden** | -0.023 | 0.237 | -0.011 | 0.924 | **0.314** | **0.149** | **0.158** | **0.035** |
| **Recruit Dublin** | -0.162 | 0.251 | -0.059 | 0.519 | **0.320** | **0.155** | **0.126** | **0.039** |
| Recruit Hamburg | -0.223 | 0.246 | -0.100 | 0.364 | 0.216 | 0.161 | 0.099 | 0.178 |
| **Recruit London** | 0.202 | 0.240 | 0.084 | 0.399 | **0.341** | **0.140** | **0.171** | **0.015** |
| Recruit Mannheim | -0.188 | 0.253 | -0.079 | 0.458 | 0.009 | 0.167 | 0.004 | 0.957 |
| Recruit Nottingham | -0.035 | 0.235 | -0.017 | 0.882 | 0.129 | 0.148 | 0.063 | 0.385 |
| Recruit Paris | -0.231 | 0.245 | -0.109 | 0.346 | 0.078 | 0.160 | 0.037 | 0.627 |
| Age 14 PDS Mean | -0.147 | 0.085 | -0.104 | 0.082 | 0.034 | 0.089 | 0.019 | 0.706 |
| **Age 14 Psych Diag** | **0.347** | **0.155** | **0.131** | **0.025** | **0.706** | **0.115** | **0.331** | **<0.001** |
| **Neg LE < age 14** | **0.073** | **0.020** | **0.236** | **<0.001** | **0.049** | **0.015** | **0.188** | **0.001** |
| Total child trauma | 0.011 | 0.007 | 0.095 | 0.129 | 0.009 | 0.005 | 0.102 | 0.054 |
| Age 14 PP ~ |  |  |  |  |  |  |  |  |
| Age 14 SE Stress | -0.013 | 0.045 | -0.021 | 0.764 | 0.015 | 0.040 | 0.024 | 0.707 |
| Age 14 Fam Supp | 0.001 | 0.029 | 0.003 | 0.970 | -0.002 | 0.028 | -0.004 | 0.951 |
| Recruit Dresden | 0.230 | 0.176 | 0.136 | 0.190 | **0.282** | **0.135** | **0.177** | **0.037** |
| Recruit Dublin | -0.199 | 0.209 | -0.088 | 0.342 | -0.131 | 0.159 | -0.064 | 0.409 |
| Recruit Hamburg | -0.040 | 0.185 | -0.022 | 0.830 | 0.088 | 0.148 | 0.050 | 0.551 |
| Recruit London | -0.137 | 0.186 | -0.069 | 0.462 | 0.236 | 0.141 | 0.148 | 0.093 |
| Recruit Mannheim | 0.039 | 0.199 | 0.020 | 0.844 | 0.285 | 0.148 | 0.161 | 0.054 |
| Recruit Nottingham | -0.077 | 0.174 | -0.044 | 0.660 | -0.009 | 0.143 | -0.006 | 0.947 |
| **Recruit Paris** | **-0.557** | **0.194** | **-0.318** | **0.004** | **-0.342** | **0.162** | **-0.204** | **0.035** |
| Age 14 PDS Mean | -0.131 | 0.074 | -0.112 | 0.075 | 0.006 | 0.081 | 0.004 | 0.944 |
| **Age 14 Psych Diag** | **0.528** | **0.137** | **0.241** | **<0.001** | **0.471** | **0.107** | **0.275** | **<0.001** |
| **Neg LE < age 14** | **0.036** | **0.017** | **0.142** | **0.028** | **0.040** | **0.013** | **0.190** | **0.003** |
| Total child trauma | 0.008 | 0.006 | 0.083 | 0.218 | 0.010 | 0.005 | 0.137 | 0.038 |
| Age 14 Amyg vol ~ |  |  |  |  |  |  |  |  |
| Age 14 SE Stress | 0.002 | 0.014 | 0.008 | 0.888 | -0.003 | 0.013 | -0.010 | 0.841 |
| Age 14 Fam Supp | 0.001 | 0.010 | 0.003 | 0.952 | 0.001 | 0.009 | 0.004 | 0.932 |
| Recruit Dresden | -0.005 | 0.062 | -0.008 | 0.934 | 0.073 | 0.042 | 0.112 | 0.080 |
| **Recruit Dublin** | 0.029 | 0.070 | 0.032 | 0.679 | **0.098** | **0.045** | **0.117** | **0.031** |
| Recruit Hamburg | 0.098 | 0.062 | 0.134 | 0.114 | 0.056 | 0.046 | 0.078 | 0.226 |
| Recruit London | -0.084 | 0.069 | -0.107 | 0.221 | **-0.141** | **0.044** | **-0.216** | **0.001** |
| Recruit Mannheim | -0.012 | 0.063 | -0.015 | 0.851 | -0.067 | 0.046 | -0.093 | 0.144 |
| **Recruit Nottingham** | **0.173** | **0.064** | **0.254** | **0.007** | **0.175** | **0.044** | **0.261** | **<0.001** |
| Recruit Paris | 0.033 | 0.066 | 0.047 | 0.622 | 0.067 | 0.045 | 0.097 | 0.138 |
| **Age 14 PDS Mean** | 0.028 | 0.024 | 0.061 | 0.248 | **0.067** | **0.029** | **0.118** | **0.019** |
| **Age 14 Psych Diag** | 0.017 | 0.048 | 0.020 | 0.718 | **-0.075** | **0.033** | **-0.106** | **0.024** |
| Neg LE < age 14 | -0.001 | 0.006 | -0.009 | 0.871 | <0.001 | 0.004 | -0.003 | 0.959 |
| Total child trauma | 0.002 | 0.002 | 0.056 | 0.357 | 0.001 | 0.002 | 0.038 | 0.460 |
| Age 14 vmPFC GMV ~ | |  |  |  |  |  |  |  |
| Age 14 SE Stress | -0.020 | 0.033 | -0.031 | 0.543 | -0.046 | 0.035 | -0.069 | 0.188 |
| Age 14 Fam Supp | 0.029 | 0.023 | 0.068 | 0.204 | -0.005 | 0.024 | -0.010 | 0.841 |
| **Recruit Dresden** | **-0.460** | **0.140** | **-0.271** | **0.001** | **-0.272** | **0.116** | **-0.161** | **0.019** |
| Recruit Dublin | 0.313 | 0.179 | 0.138 | 0.080 | **0.516** | **0.129** | **0.240** | **<0.001** |
| Recruit Hamburg | -0.195 | 0.154 | -0.106 | 0.203 | **-0.263** | **0.117** | **-0.142** | **0.025** |
| Recruit London | -0.037 | 0.149 | -0.019 | 0.802 | -0.113 | 0.109 | -0.067 | 0.299 |
| **Recruit Mannheim** | **-0.551** | **0.146** | **-0.281** | **<0.001** | **-0.624** | **0.112** | **-0.333** | **<0.001** |
| Recruit Nottingham | 0.018 | 0.150 | 0.010 | 0.904 | 0.168 | 0.121 | 0.097 | 0.167 |
| Recruit Paris | -0.094 | 0.160 | -0.053 | 0.560 | -0.071 | 0.125 | -0.040 | 0.569 |
| Age 14 PDS Mean | -0.012 | 0.065 | -0.010 | 0.854 | -0.026 | 0.066 | -0.018 | 0.695 |
| Age 14 Psych Diag | -0.191 | 0.113 | -0.087 | 0.091 | -0.086 | 0.088 | -0.047 | 0.333 |
| Neg LE < age 14 | 0.004 | 0.014 | 0.014 | 0.797 | -0.006 | 0.012 | -0.025 | 0.637 |
| Total child trauma | 0.001 | 0.006 | 0.013 | 0.824 | 0.005 | 0.004 | 0.063 | 0.251 |
| ΔES ~ |  |  |  |  |  |  |  |  |
| **Age 14 ES** | **-0.677** | **0.136** | **-0.526** | **<0.001** | **-0.254** | **0.123** | **-0.216** | **0.039** |
| Age 14 PP | 0.262 | 0.166 | 0.168 | 0.114 | -0.258 | 0.159 | -0.176 | 0.105 |
| Age 14 Amyg vol | 0.362 | 0.255 | 0.092 | 0.156 | 0.126 | 0.307 | 0.035 | 0.682 |
| Age 14 vmPFC GMV | -0.062 | 0.104 | -0.040 | 0.549 | -0.010 | 0.128 | -0.007 | 0.941 |
| Age 14 SE Stress | 0.009 | 0.052 | 0.009 | 0.862 | 0.090 | 0.047 | 0.098 | 0.059 |
| Age 14 Fam Supp | 0.039 | 0.035 | 0.060 | 0.262 | 0.019 | 0.035 | 0.029 | 0.593 |
| Recruit Dresden | -0.248 | 0.259 | -0.094 | 0.339 | -0.020 | 0.185 | -0.008 | 0.915 |
| Recruit Dublin | 0.493 | 0.267 | 0.139 | 0.065 | -0.132 | 0.192 | -0.044 | 0.494 |
| Recruit Hamburg | 0.202 | 0.240 | 0.070 | 0.399 | -0.120 | 0.191 | -0.046 | 0.531 |
| **Recruit London** | **0.529** | **0.230** | **0.170** | **0.021** | 0.150 | 0.174 | 0.064 | 0.390 |
| Recruit Mannheim | -0.010 | 0.256 | -0.003 | 0.970 | 0.255 | 0.181 | 0.098 | 0.158 |
| Recruit Nottingham | 0.371 | 0.225 | 0.137 | 0.099 | -0.049 | 0.180 | -0.020 | 0.787 |
| Recruit Paris | 0.104 | 0.248 | 0.038 | 0.677 | -0.112 | 0.196 | -0.046 | 0.566 |
| Age 14 PDS Mean | 0.057 | 0.103 | 0.031 | 0.582 | -0.092 | 0.112 | -0.045 | 0.414 |
| Age 14 Psych Diag | 0.026 | 0.179 | 0.008 | 0.886 | 0.156 | 0.141 | 0.062 | 0.267 |
| Neg LE < age 14 | -0.034 | 0.024 | -0.084 | 0.165 | 0.014 | 0.018 | 0.045 | 0.440 |
| **Neg LE > age 14** | **0.045** | **0.019** | **0.134** | **0.016** | **0.079** | **0.016** | **0.290** | **<0.001** |
| **Total child trauma** | **0.040** | **0.009** | **0.274** | **<0.001** | **0.016** | **0.006** | **0.145** | **0.005** |
| ΔPP ~ |  |  |  |  |  |  |  |  |
| Age 14 ES | 0.025 | 0.075 | 0.033 | 0.739 | 0.077 | 0.072 | 0.089 | 0.287 |
| **Age 14 PP** | **-0.630** | **0.111** | **-0.687** | **<0.001** | **-0.783** | **0.109** | **-0.730** | **<0.001** |
| Age 14 Amyg vol | 0.207 | 0.147 | 0.089 | 0.161 | 0.068 | 0.185 | 0.026 | 0.714 |
| Age 14 vmPFC GMV | -0.014 | 0.059 | -0.016 | 0.807 | 0.011 | 0.084 | 0.011 | 0.899 |
| Age 14 SE Stress | 0.035 | 0.034 | 0.059 | 0.299 | 0.043 | 0.043 | 0.063 | 0.318 |
| Age 14 Fam Supp | -0.009 | 0.020 | -0.023 | 0.662 | -0.013 | 0.026 | -0.028 | 0.610 |
| Recruit Dresden | 0.024 | 0.161 | 0.015 | 0.884 | -0.013 | 0.150 | -0.008 | 0.929 |
| Recruit Dublin | 0.022 | 0.181 | 0.011 | 0.904 | -0.041 | 0.162 | -0.019 | 0.801 |
| Recruit Hamburg | -0.020 | 0.153 | -0.012 | 0.896 | -0.195 | 0.158 | -0.103 | 0.216 |
| Recruit London | 0.070 | 0.162 | 0.039 | 0.664 | 0.065 | 0.145 | 0.038 | 0.654 |
| Recruit Mannheim | -0.193 | 0.179 | -0.107 | 0.282 | -0.028 | 0.165 | -0.015 | 0.865 |
| Recruit Nottingham | 0.024 | 0.158 | 0.015 | 0.878 | 0.079 | 0.149 | 0.045 | 0.597 |
| Recruit Paris | 0.219 | 0.162 | 0.136 | 0.176 | 0.059 | 0.158 | 0.033 | 0.706 |
| **Age 14 PDS Mean** | **0.158** | **0.062** | **0.147** | **0.011** | -0.093 | 0.085 | -0.063 | 0.273 |
| Age 14 Psych Diag | -0.079 | 0.111 | -0.039 | 0.474 | 0.154 | 0.112 | 0.084 | 0.171 |
| **Neg LE < age 14** | **-0.033** | **0.016** | **-0.140** | **0.036** | -0.004 | 0.013 | -0.019 | 0.751 |
| **Neg LE > age 14** | 0.015 | 0.013 | 0.077 | 0.238 | **0.031** | **0.012** | **0.154** | **0.012** |
| **Total child trauma** | **0.021** | **0.006** | **0.246** | **<0.001** | 0.008 | 0.005 | 0.095 | 0.137 |
| ΔAmyg vol ~ |  |  |  |  |  |  |  |  |
| Age 14 ES | -0.001 | 0.026 | -0.005 | 0.962 | -0.022 | 0.018 | -0.106 | 0.241 |
| Age 14 PP | -0.002 | 0.032 | -0.006 | 0.955 | 0.011 | 0.026 | 0.043 | 0.678 |
| **Age 14 Amyg vol** | **-0.290** | **0.046** | **-0.391** | **<0.001** | **-0.229** | **0.047** | **-0.370** | **<0.001** |
| Age 14 vmPFC GMV | -0.032 | 0.019 | -0.109 | 0.091 | -0.011 | 0.018 | -0.046 | 0.533 |
| Age 14 SE Stress | -0.009 | 0.011 | -0.046 | 0.441 | 0.015 | 0.009 | 0.096 | 0.108 |
| **Age 14 Fam Supp** | **-0.017** | **0.007** | **-0.138** | **0.023** | 0.004 | 0.005 | 0.040 | 0.421 |
| **Recruit Dresden** | **-0.200** | **0.045** | **-0.404** | **<0.001** | **-0.147** | **0.031** | **-0.363** | **<0.001** |
| Recruit Dublin | -0.096 | 0.053 | -0.144 | 0.074 | -0.028 | 0.037 | -0.054 | 0.461 |
| **Recruit Hamburg** | **-0.112** | **0.044** | **-0.208** | **0.011** | -0.046 | 0.034 | -0.102 | 0.176 |
| **Recruit London** | **-0.140** | **0.046** | **-0.241** | **0.002** | -0.044 | 0.034 | -0.109 | 0.195 |
| **Recruit Mannheim** | **-0.205** | **0.046** | **-0.357** | **<0.001** | **-0.163** | **0.039** | **-0.363** | **<0.001** |
| **Recruit Nottingham** | **-0.224** | **0.042** | **-0.442** | **<0.001** | **-0.114** | **0.034** | **-0.275** | **0.001** |
| Recruit Paris | -0.081 | 0.045 | -0.158 | 0.072 | -0.012 | 0.032 | -0.029 | 0.706 |
| Age 14 PDS Mean | -0.030 | 0.017 | -0.087 | 0.071 | 0.009 | 0.021 | 0.027 | 0.650 |
| Age 14 Psych Diag | -0.030 | 0.037 | -0.046 | 0.423 | 0.036 | 0.027 | 0.083 | 0.182 |
| Neg LE < age 14 | -0.004 | 0.004 | -0.060 | 0.277 | -0.002 | 0.003 | -0.034 | 0.490 |
| Neg LE > age 14 | -0.004 | 0.004 | -0.067 | 0.343 | -0.005 | 0.004 | -0.106 | 0.181 |
| Total child trauma | -0.003 | 0.002 | -0.105 | 0.068 | 0.001 | 0.001 | 0.043 | 0.385 |
| ΔvmPFC GMV ~ |  |  |  |  |  |  |  |  |
| **Age 14 ES** | -0.032 | 0.053 | -0.045 | 0.551 | **-0.089** | **0.044** | **-0.167** | **0.044** |
| Age 14 PP | -0.018 | 0.072 | -0.022 | 0.797 | 0.095 | 0.074 | 0.144 | 0.194 |
| Age 14 Amyg vol | 0.113 | 0.121 | 0.052 | 0.349 | 0.039 | 0.119 | 0.024 | 0.746 |
| **Age 14 vmPFC GMV** | **-0.503** | **0.054** | **-0.588** | **<0.001** | **-0.381** | **0.059** | **-0.606** | **<0.001** |
| Age 14 SE Stress | 0.001 | 0.021 | 0.001 | 0.969 | -0.004 | 0.019 | -0.010 | 0.825 |
| Age 14 Fam Supp | -0.017 | 0.018 | -0.046 | 0.370 | 0.001 | 0.015 | 0.005 | 0.929 |
| **Recruit Dresden** | **0.362** | **0.096** | **0.250** | **<0.001** | **0.202** | **0.077** | **0.190** | **0.009** |
| **Recruit Dublin** | **0.308** | **0.111** | **0.158** | **0.005** | **0.264** | **0.092** | **0.195** | **0.004** |
| Recruit Hamburg | 0.002 | 0.093 | 0.001 | 0.985 | **0.171** | **0.080** | **0.146** | **0.034** |
| Recruit London | 0.162 | 0.094 | 0.095 | 0.083 | **0.222** | **0.073** | **0.210** | **0.002** |
| Recruit Mannheim | -0.120 | 0.103 | -0.072 | 0.245 | 0.027 | 0.092 | 0.023 | 0.774 |
| Recruit Nottingham | 0.057 | 0.089 | 0.038 | 0.525 | 0.136 | 0.081 | 0.125 | 0.092 |
| **Recruit Paris** | **-0.489** | **0.098** | **-0.325** | **<0.001** | **-0.201** | **0.083** | **-0.180** | **0.015** |
| Age 14 PDS Mean | -0.042 | 0.045 | -0.042 | 0.355 | 0.061 | 0.049 | 0.066 | 0.219 |
| Age 14 Psych Diag | 0.118 | 0.095 | 0.063 | 0.217 | -0.002 | 0.068 | -0.002 | 0.975 |
| **Neg LE < age 14** | -0.006 | 0.011 | -0.029 | 0.558 | **0.014** | **0.007** | **0.102** | **0.042** |
| **Neg LE > age 14** | -0.017 | 0.011 | -0.091 | 0.112 | **-0.021** | **0.009** | **-0.169** | **0.016** |
| Total child trauma | 0.002 | 0.003 | 0.026 | 0.533 | <0.001 | 0.003 | -0.002 | 0.974 |
|  |  |  |  |  |  |  |  |  |
| Covariances: |  |  |  |  |  |  |  |  |
|  | Est | SE | Std.all | *p* | Est | SE | Std.all | *p* |
| **Age 14 Loner ~~**  **Age 19 Loner** | **0.212** | **0.082** | **0.197** | **0.009** | **0.234** | **0.082** | **0.218** | **0.004** |
| Age 14 Friend ~~  Age 19 Friend | 0.011 | 0.066 | 0.023 | 0.865 | -0.261 | 0.174 | -0.238 | 0.133 |
| **Age 14 Popular ~~**  **Age 19 Popular** | **0.140** | **0.066** | **0.179** | **0.035** | **0.166** | **0.066** | **0.213** | **0.012** |
| **Age 14 Bullied ~~**  **Age 19 Bullied** | 0.149 | 0.121 | 0.202 | 0.218 | **0.416** | **0.149** | **0.506** | **0.005** |
| **Age 14 Old Best ~~**  **Age 19 Old Best** | **0.344** | **0.081** | **0.352** | **<0.001** | **0.401** | **0.083** | **0.367** | **<0.001** |
| **Age 14 Worries ~~**  **Age 19 Worries** | **0.277** | **0.097** | **0.297** | **0.004** | **0.277** | **0.075** | **0.297** | **<0.001** |
| **Age 14 Clingy ~~**  **Age 19 Clingy** | **0.298** | **0.068** | **0.381** | **<0.001** | **0.398** | **0.059** | **0.508** | **<0.001** |
| Age 14 Somatic ~~  Age 19 Somatic | 0.139 | 0.097 | 0.141 | 0.151 | **0.368** | **0.072** | **0.374** | **<0.001** |
| Age 14 Unhappy ~~  Age 19 Unhappy | 0.004 | 0.136 | 0.005 | 0.979 | -0.085 | 0.094 | -0.112 | 0.362 |
| **Age 14 Afraid ~~**  **Age 19 Afraid** | **0.344** | **0.105** | **0.457** | **0.001** | **0.260** | **0.074** | **0.345** | **<0.001** |
| **Age 14 L Amyg ~~**  **Age 19 L Amyg** | **0.005** | **0.002** | **0.414** | **0.001** | **0.005** | **0.001** | **0.390** | **<0.001** |
| **Age 14 R Amyg ~~**  **Age 19 R Amyg** | **0.008** | **0.002** | **0.414** | **<0.001** | **0.007** | **0.002** | **0.349** | **<0.001** |
| **Age 14 L vmPFC ~~**  **Age 19 L vmPFC** | **0.148** | **0.021** | **0.617** | **<0.001** | **0.109** | **0.017** | **0.453** | **<0.001** |
| **Age 14 R vmPFC ~~**  **Age 19 R vmPFC** | **0.076** | **0.018** | **0.447** | **<0.001** | **0.075** | **0.016** | **0.444** | **<0.001** |
| **Age 14 PP ~~**  **Age 14 ES** | **0.237** | **0.038** | **0.593** | **<0.001** | **0.136** | **0.025** | **0.460** | **<0.001** |
| Age 14 PP ~~  Age 14 Amyg vol | 0.002 | 0.009 | 0.019 | 0.776 | 0.001 | 0.007 | 0.010 | 0.885 |
| Age 14 PP ~~  Age 14 vmPFC GMV | 0.016 | 0.022 | 0.050 | 0.454 | -0.028 | 0.017 | -0.112 | 0.092 |
| Age 14 ES ~~  Age 14 Amyg vol | 0.010 | 0.011 | 0.056 | 0.381 | 0.013 | 0.008 | 0.101 | 0.086 |
| Age 14 ES ~~  Age 14 vmPFC GMV | -0.039 | 0.026 | -0.094 | 0.131 | -0.003 | 0.020 | -0.009 | 0.879 |
| **Age 14 Amyg vol ~~**  **Age 14 vmPFC GMV** | **0.060** | **0.008** | **0.435** | **<0.001** | **0.056** | **0.007** | **0.508** | **<0.001** |
| **ΔPP ~~**  **ΔES** | **0.171** | **0.033** | **0.623** | **<0.001** | **0.230** | **0.034** | **0.708** | **<0.001** |
| ΔAmyg vol ~~  ΔPP | 0.004 | 0.005 | 0.086 | 0.357 | -0.001 | 0.005 | -0.029 | 0.758 |
| **ΔvmPFC GMV ~~**  **ΔPP** | **0.026** | **0.010** | **0.215** | **0.011** | -0.017 | 0.012 | -0.155 | 0.146 |
| ΔAmyg vol ~~  ΔES | 0.014 | 0.008 | 0.122 | 0.091 | 0.005 | 0.007 | 0.053 | 0.513 |
| **ΔvmPFC GMV ~~**  **ΔES** | **0.049** | **0.020** | **0.166** | **0.015** | 0.014 | 0.016 | 0.077 | 0.378 |
| **ΔAmyg vol ~~**  **ΔvmPFC GMV** | **0.026** | **0.004** | **0.497** | **<0.001** | **0.016** | **0.003** | **0.553** | **<0.001** |

*Note.* Ages are in years. Est = estimate, SE = standard error, Std.all = standardised estimate (both latent and observed variables are standardised to have a variance of 1), ES = emotional symptoms, SE = socioeconomic, Fam Supp = family support, Recruit = Recruitment Centre (dummy coded variables with baseline of ‘Berlin’), PDS = Pubertal Development Scale, Psych Diag = psychiatric diagnosis, Neg LE = negative life events, PP = peer problems, Amyg vol = amygdala volume, vmPFC GMV = ventromedial prefrontal grey matter volume,
Δ = change, ~ = predicted by, ~~ = covariance.
Statistically significant parameters at the p < 0.05 in bold.

**Online Resource 10**

*Measurement invariance models for the family support latent variable at age 14 years between sex*

|  | **Robust model fit** | | |  |  | **Robust χ^2^  difference test** | | |
| --- | --- | --- | --- | --- | --- | --- | --- | --- |
| **Model** | χ^2^ | df | *p* | CFI | RMSEA | χ^2^ | df | *p* |
| Configural Invariance | 3.236 | 4 | 0.519 | 1.000 | 0.001 [0.001-0.063] | - | - | *-* |
| Full Loading Invariance | 8.506 | 7 | 0.290 | 0.998 | 0.021  [0.001-0.063] | 4.614 | 3 | 0.202 |
| Full Intercept Invariance | 16.754 | 10 | 0.080 | 0.993 | 0.038  [0.001-0.068] | 6.760 | 3 | 0.080 |
| Full Residual Invariance | 133.471 | 94 | 0.005 | 0.984 | 0.030 [0.017-0.041] | 4.830 | 4 | 0.305 |

**Online Resource 11**

*Statistically significant parameters of interest from the family support (latent variable) and amygdala volume latent change score model*

|  | **Males (n = 448)** | | | |  | **Females (n = 509)** | | | | |
| --- | --- | --- | --- | --- | --- | --- | --- | --- | --- | --- |
| **Parameter** | Est | SE | Std.all | *p* |  | Est | SE | Std.all | *p* |  |
| **Family Support Age 14 Latent Variable** |  |  |  |  |  |  |  |  |  |  |
| Praised and rewarded | 1.276 | 0.126 | 0.737 | <0.001 |  | 1.276 | 0.126 | 0.721 | <0.001 |  |
| Gets love and affection | 1.215 | 0.166 | 0.852 | <0.001 |  | 1.215 | 0.166 | 0.840 | <0.001 |  |
| Gets help and support when stressed | 1.126 | 0.146 | 0.760 | <0.001 |  | 1.126 | 0.146 | 0.745 | <0.001 |  |
| Liked and respected | 1.259 | 0.192 | 0.806 | <0.001 |  | 1.259 | 0.192 | 0.792 | <0.001 |  |
| **Amygdala Volume Age 14 Latent Variable** |  |  |  |  |  |  |  |  |  |  |
| Left Amygdala Age 14 | 1.049 | 0.024 | 0.866 | <0.001 |  | 1.049 | 0.024 | 0.853 | <0.001 |  |
| Right Amygdala Age 14 | 1.103 | 0.027 | 0.840 | <0.001 |  | 1.103 | 0.027 | 0.825 | <0.001 |  |
| **Amygdala Volume Age 19 Latent Variable** |  |  |  |  |  |  |  |  |  |  |
| Left Amygdala Age 19 | 1.049 | 0.024 | 0.847 | <0.001 |  | 1.049 | 0.024 | 0.832 | <0.001 |  |
| Right Amygdala Age 19 | 1.103 | 0.027 | 0.819 | <0.001 |  | 1.103 | 0.027 | 0.802 | <0.001 |  |
| **Regression Parameters** |  |  |  |  |  |  |  |  |  |  |
| Coupling: Family Support → ΔAmygdala | -0.340 | 0.043 | -0.459 | 0.005 |  | 0.010 | 0.009 | 0.072 | 0.232 |  |

*Note.* Model fit (Robust χ2 (185) = 321.771, p < 0.001; CFI = 0.918; RMSEA = 0.039, 90% CI = [0.032, 0.046])

**Online Resource 12**

*Covariate-corrected quadvariate model with statistically significant parameters of interest, controlling for participants with a mood or anxiety disorder instead of any psychiatric diagnosis (n = 957)*

|  | **Males (n = 448)** | | | |  | **Females (n = 509)** | | | |
| --- | --- | --- | --- | --- | --- | --- | --- | --- | --- |
| **Statistically significant parameters of interest** | Est | SE | Std.all | *p* |  | Est | SE | Std.all | *p* |
| Coupling: Age 14 ES → ΔvmPFC GMV | -0.037 | 0.057 | -0.049 | 0.516 |  | -0.082 | 0.047 | -0.146 | 0.084 |
| Coupling: Age 14 Family support → ΔAmyg vol | **-0.016** | **0.007** | **-0.135** | **0.027** |  | 0.004 | 0.005 | 0.038 | 0.446 |
| Correlated change: ΔPP and ΔES | **0.163** | **0.032** | **0.604** | **<0.001** |  | **0.226** | **0.033** | **0.709** | **<0.001** |
| Correlated change: ΔAmyg vol and ΔvmPFC GMV | **0.026** | **0.004** | **0.486** | **<0.001** |  | **0.016** | **0.003** | **0.560** | **<0.001** |
| Correlated change: ΔPP and ΔvmPFC | **0.027** | **0.011** | **0.210** | **0.014** |  | -0.016 | 0.012 | -0.145 | 0.176 |
| Correlated change: ΔES and ΔvmPFC | **0.056** | **0.021** | **0.181** | **0.008** |  | 0.015 | 0.017 | 0.076 | 0.384 |

*Note.* Ages are in years. PP = peer problems, ES = emotional symptoms, vmPFC = ventromedial prefrontal cortex, GMV = grey matter volume, Amyg vol = amygdala volume, Δ = change.
Model fit: (χ2 (1248) = 1605.690, p < 0.001; CFI = 0.923; RMSEA = 0.024, 90% CI = [0.021, 0.028])
